# Supplementary material for: Dynamical interplay between the human high-affinity copper transporter hCtr1 and its cognate metal ion
Source: Biophys J. 2022 Feb 22;121(7):1194–204. doi: 10.1016/j.bpj.2022.02.033 (PMC9034245; doi:10.1016/j.bpj.2022.02.033)
Supplement: Document S1. Figures S1–S11 and Scheme S1 [file mmc1.pdf]

Biophysical Journal, Volume *121*

Supplemental information

Dynamical interplay between the human high-affinity copper transporter hCtr1 and its cognate metal ion

Gulshan Walke, Jana Aupič, Hadeel Kashoua, Pavel Janoš, Shelly Meron, Yulia Shenberger, Zena Qasem, Lada Gevorkyan-Airapetov, Alessandra Magistrato, and Sharon Ruthstein

Dynamical interplay between the human high-affinity copper transporter hCtr1 and its cognate metal ion

Gulshan Walke[a,#], Jana Aupič[b,#], Hadeel Kashoua[a], Pavel Janoš[b], Shelly Meron[a], Yulia Shenberger[a], Zena Qasem[a], Lada Gevorkyan-Airapetov[a], Alessandra Magistrato[b,*], Sharon Ruthstein[a,*]

[a] Dr. Gulshan Walke, Ms. Hadeel Kashoua, Ms. Shelly Meron, Dr. Yulia Shenberger, Ms. Zena Qasem, Dr. Lada Gevorkyan-Airapetov, Prof. Sharon Ruthstein
Department of Chemistry and the Institute of Nanotechnology and Advanced Materials (BINA), Bar-Ilan University, Ramat-Gan, Israel, 5290002
E-mail: Sharon.ruthstein@biu.ac.il

[b] Dr. Jana Aupic, Dr. Pavel Janos, Prof. Alessandra Magistrato
Department National Research Council of Italy (CNR) - Institute of Material (IOM) c/o International School for Advanced Studies (SISSA), via Bonomea 265, 34136 Trieste, Italy
Email: alessandra.magistrato@sissa.it

[#] Equal contributions
[*] Corresponding authors

Supporting Information

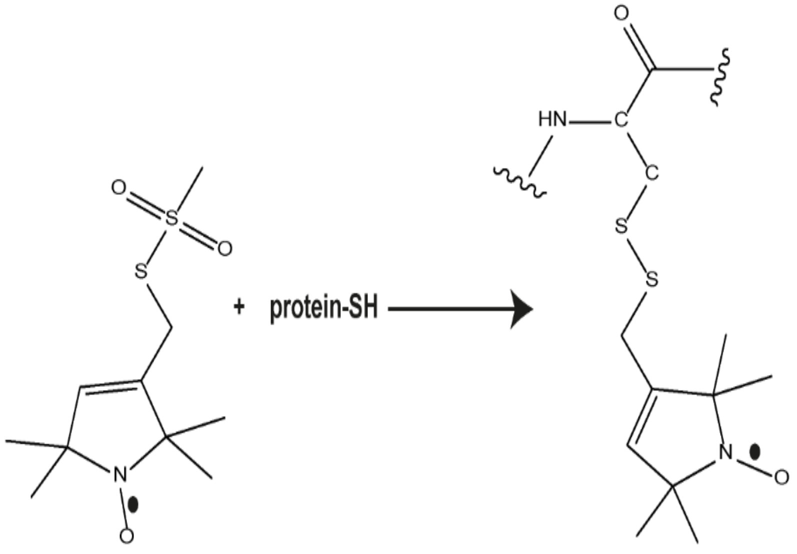

Scheme S1: Spin labeling scheme of cysteine residues with methanesulfonothioate (MTSSL) nitroxide radical.

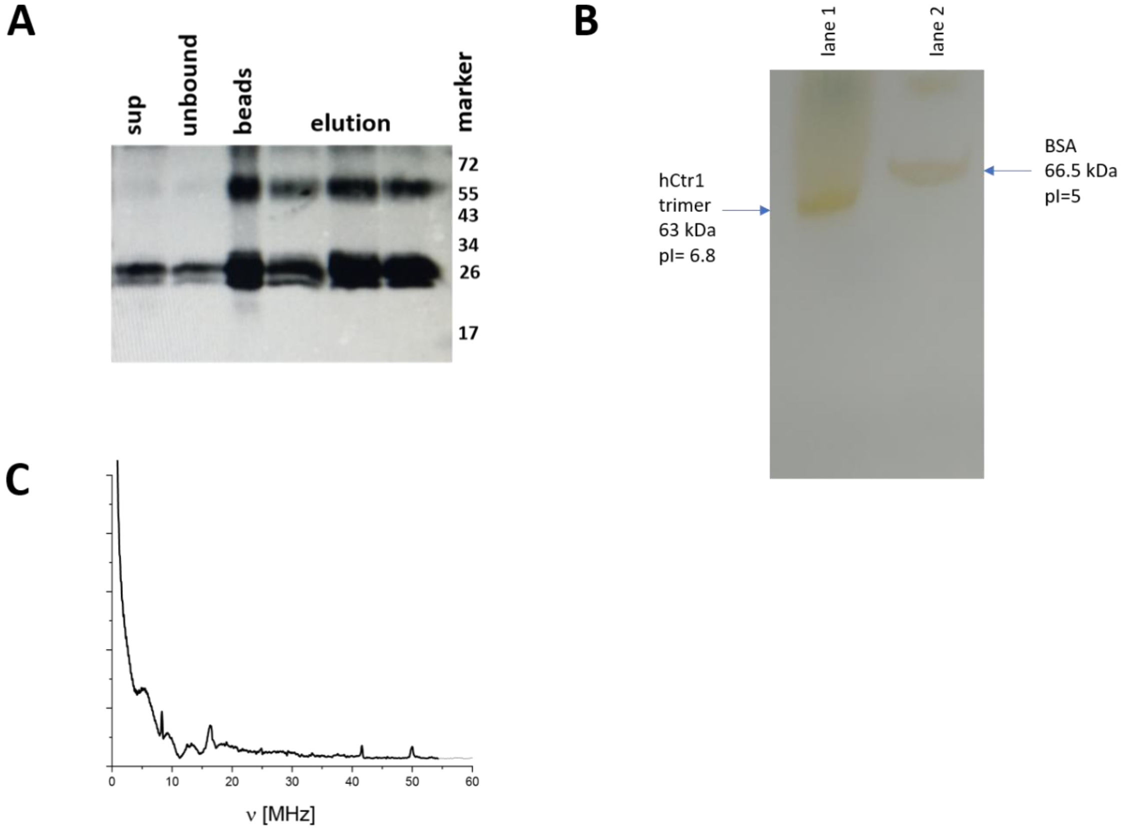

Figure S1: A. Western blot analysis of hCtr1 protein fractions probed. **B. Native-polyacrylamide gel electrophoresis.** Native-PAGE 12% glycine pH 8.5. Lane 1, the purified hCtr1 protein (MW of the monomer 21kDa, pI 6.8). Lane 2, the custom BSA protein (MW of the monomer 66.5 kDa, pI 5). **C**. **Q-band 3P-ESEEM FT spectrum** of Cu(II) hCtr1 (1:1 ratio). The experiment was carried out at 33.72 GHz, 11640 G, τ=180 ns, dt= 12ns. 600 points, Temp. =20 K.

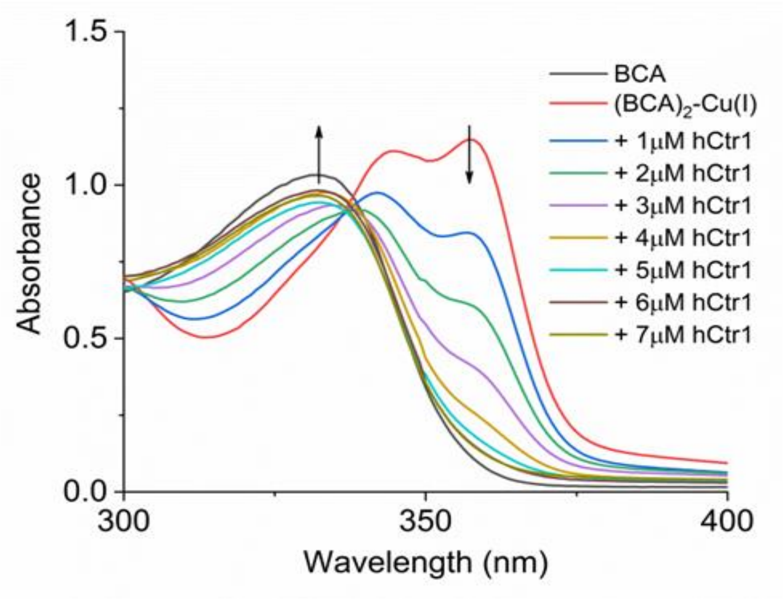

Figure S2. The UV-Vis spectra of the titration of hCtr1 to $(BCA)_2$-Cu(I) solution in UV region. The concentration of BCA and Cu(I) was 60 μM and 25 μM respectively to form 25 μM $(BCA)_2$-Cu(I) complex.

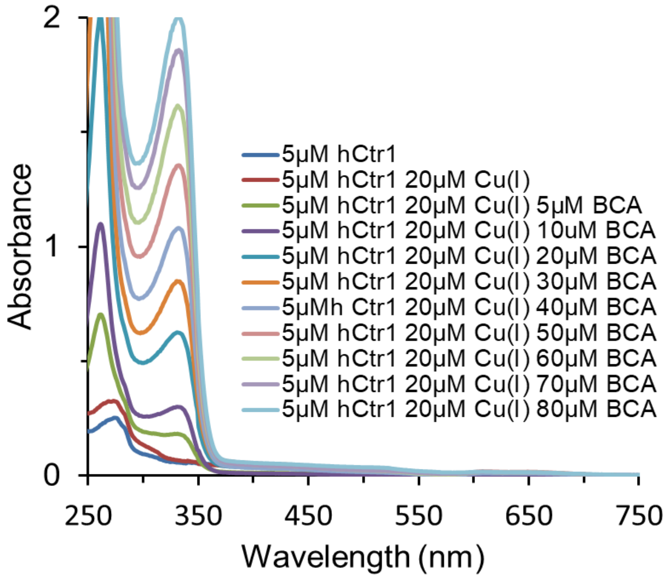

Figure S3. The UV-Vis spectra of the titration of BCA to hCtr1-Cu(I) solution in UV region.

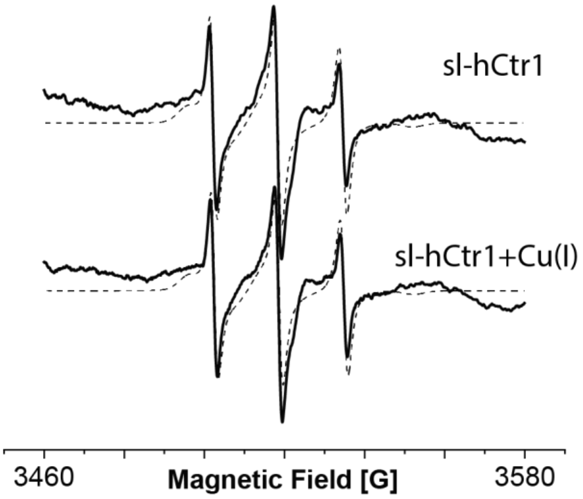

Figure S4. Room temperature X-band CW EPR spectrum of spin labelled (sl) wild type hCtr1 protein (named sl-hCtr1) in the absence and in the presence of Cu(I). The data was simulated using the easyspin program implemented in MATLAB, with g= [2.0087 2.006 2.0022], and A=[25 25 88] MHz.

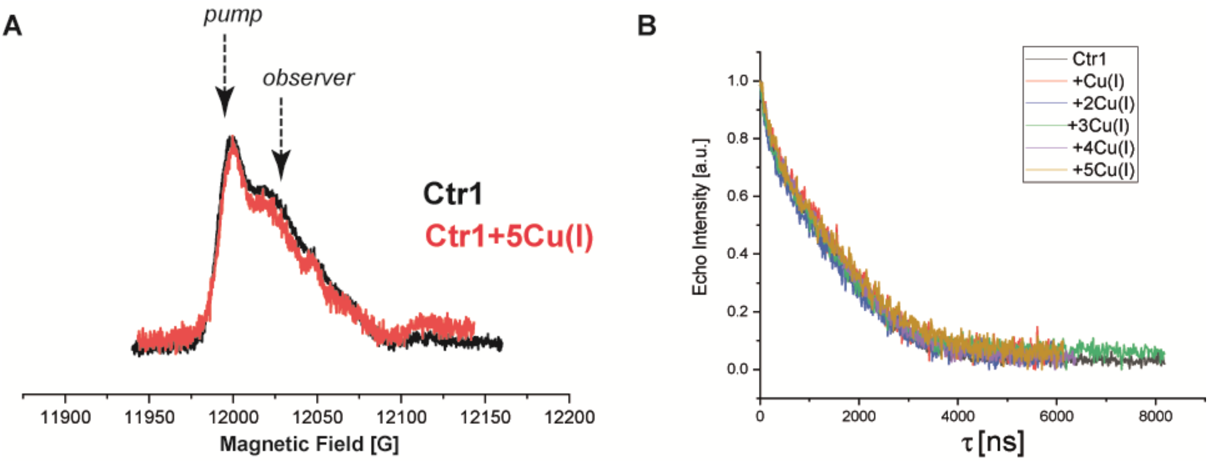

Figure S5. A. Two pulsed field sweep Q-band EPR spectra (50K) of wild-type spin-labeled hCtr1 in the presence and absence of Cu(I). **B**. two-pulse echo decay signals of spin-labeled hCtr1 at the absence and presence of Cu(I) ions.

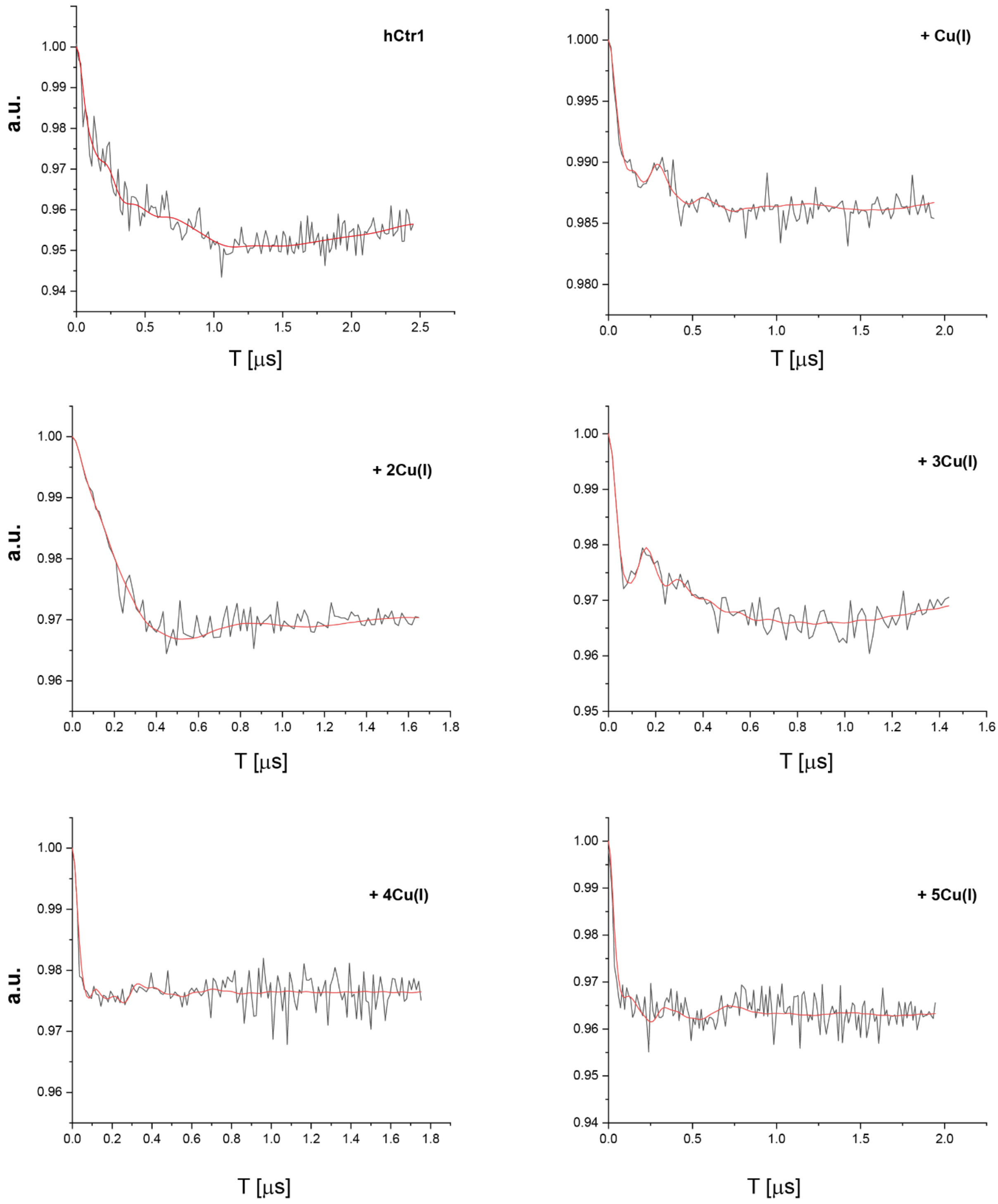

Figure S6. Q-band DEER time domain signals carried out at 50K of spin labelled wild type hCtr1 protein in the absence and in the presence of Cu(I) in Hepes buffer (0.1% Triton), pH 7.4

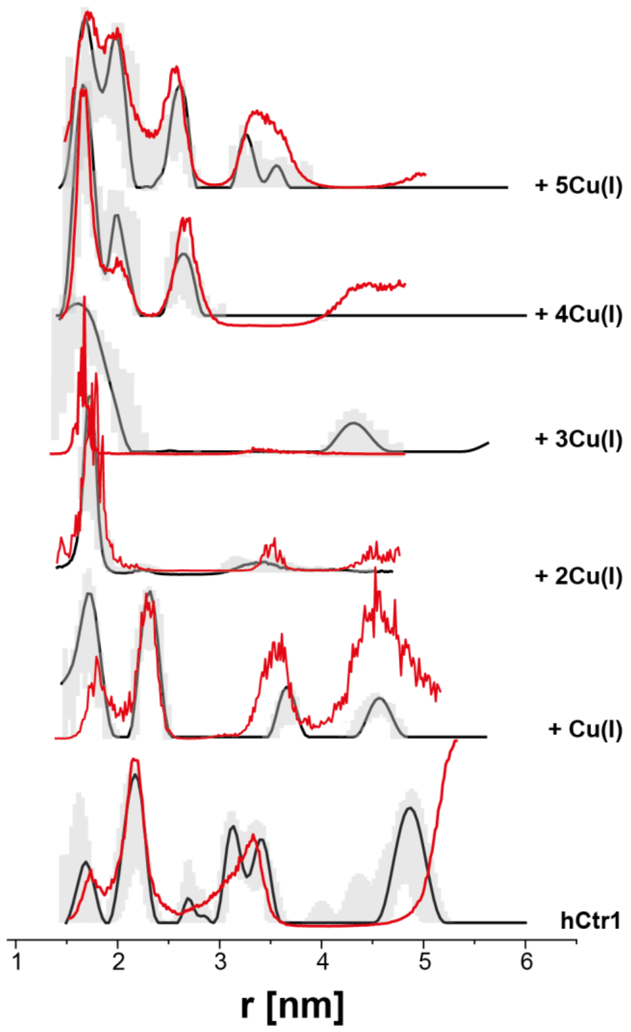

Figure S7. Q-band DEER distance distribution functions of spin labelled wild type hCtr1 protein. Black solid lines represent analysis using Tikhonov regularization, and red lines represent DeerNet analysis.

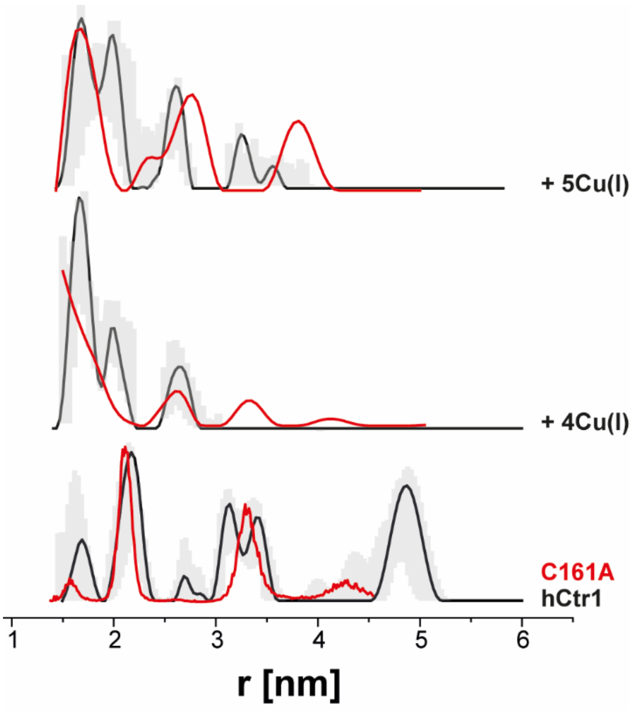

Figure S8. Q-band DEER distance distribution functions of spin labelled wild type hCtr1 protein (black solid line) and hCtr1_C161A mutant at various Cu(I) concentrations in Hepes buffer (0.1% Triton), pH 7.4.

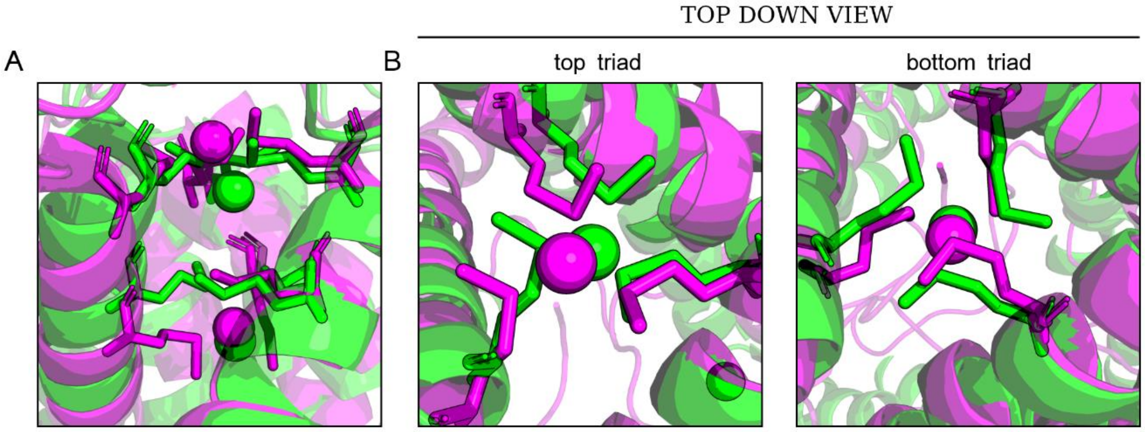

Figure S9. Comparison of the coordination geometry of Cu(I) ions bound in the selectivity filter in the crystal structure (PDBID:6m98) and in a representative structure extracted from QM/MM molecular dynamics simulation, shown in green and violet, respectively. **A**. The RMSD of the QM/MM MD optimized selectivity filter was 5.3 Å with respect to the crystal structure. RMSD was calculated using Met side chains and Cu(I) ions. **B**. Top-down view of the top and bottom Met triad (middle and right panel, repectively).

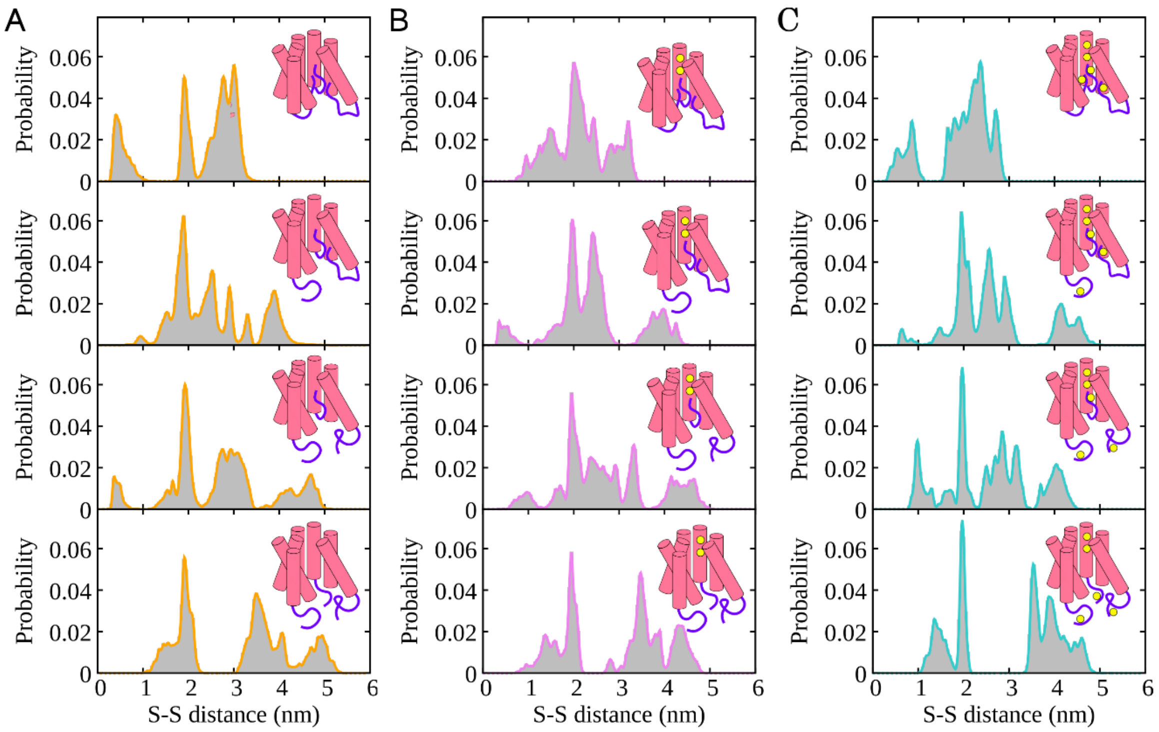

Figure S10. Distance distributions (nm) of Cys189 and Cys161 for all investigated hCtr1 models differing in the initial C-term tail conformation and Cu(I) loading. Distance distributions obtained for (**A**) apo hCtr1, (**B**) hCtr1 with two Cu(I) ions in the selectivity filter and (**C**) fully holo hCtr1 (with two Cu(I) ions in the selectivity filter and one Cu(I) in each C-terminal end). The cartoon shows the hCtr1 model with the transmembrane helices depicted in pink cylinder and each C-terminus depicted with a violet line. Copper is shown as a yellow circle.

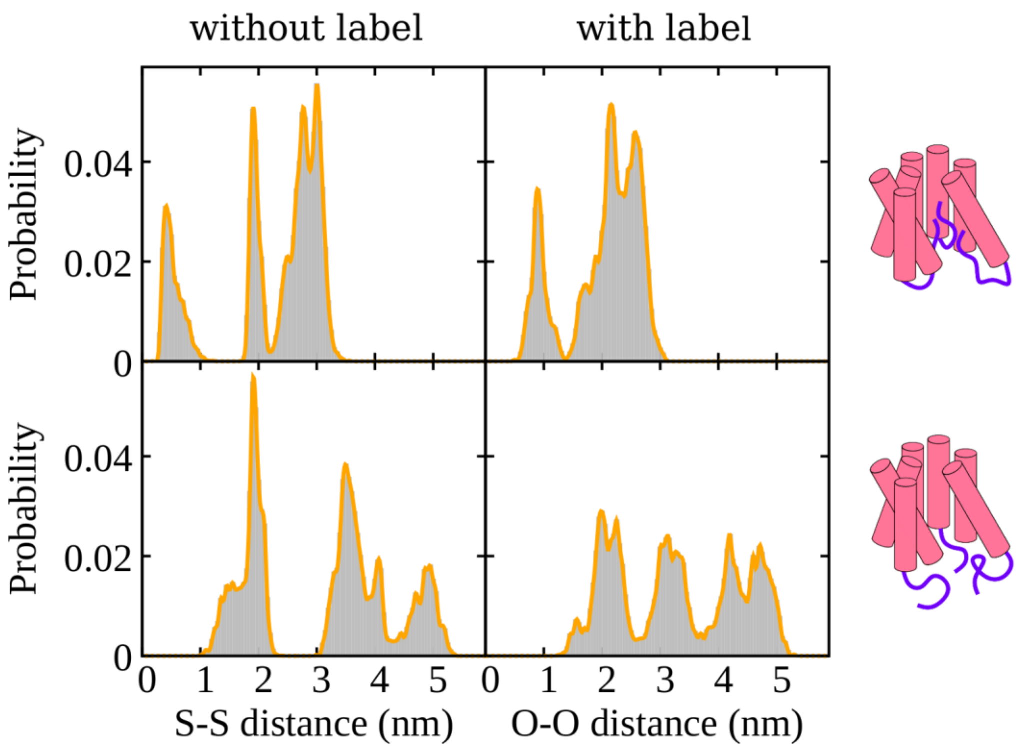

Figure S11. Impact of the MTSSL label on the distance distributions (nm) obtained from Molecular dynamics (MD) simulations. Left panels depict the distribution of distances between sulphur atoms present in Cys189 and Cys161 for the case where all three C-terminal tails are in the channel lumen (top) or cytosol (bottom). The MD simulations were performed in the absence (left panel) and in the presence of the MTSSL label attached to the Cys residues (right panel). Distance distributions were calculated between oxygen radical present in MTSSL. All simulations were performed for hCtr1 in the apo state.
