# Supplementary material for: Dynamical interplay between the human high-affinity copper transporter hCtr1 and its cognate metal ion
Source: Biophys J. 2022 Feb 22;121(7):1194–204. doi: 10.1016/j.bpj.2022.02.033 (PMC9034245; doi:10.1016/j.bpj.2022.02.033)
Supplement: Document S2. Article plus supporting material [file mmc2.pdf]

Biophysical Journal
Article

Biophysical Society

Dynamical interplay between the human high-affinity copper transporter hCtr1 and its cognate metal ion

Gulshan Walke,[1,3] Jana Aupič,[2,3] Hadeel Kashoua,[1] Pavel Janoš,[2] Shelly Meron,[1] Yulia Shenberger,[1] Zena Qasem,[1] Lada Gevorkyan-Airapetov,[1] Alessandra Magistrato,[2,*] and Sharon Ruthstein[1,*]
[1]Department of Chemistry and the Institute of Nanotechnology and Advanced Materials (BINA), Bar-Ilan University, Ramat-Gan, Israel and [2]Department National Research Council of Italy (CNR) - Institute of Material (IOM) c/o International School for Advanced Studies (SISSA), Trieste, Italy

ABSTRACT Abnormal cellular copper levels have been clearly implicated in genetic diseases, cancer, and neurodegeneration. Ctr1, a high-affinity copper transporter, is a homotrimeric integral membrane protein that provides the main route for cellular copper uptake. Together with a sophisticated copper transport system, Ctr1 regulates Cu(I) metabolism in eukaryotes. Despite its pivotal role in normal cell function, the molecular mechanism of copper uptake and transport via Ctr1 remains elusive. In this study, electron paramagnetic resonance (EPR), UV-visible spectroscopy, and all-atom simulations were employed to explore Cu(I) binding to full-length human Ctr1 (hCtr1), thereby elucidating how metal binding at multiple distinct sites affects the hCtr1 conformational dynamics. We demonstrate that each hCtr1 monomer binds up to five Cu(I) ions and that progressive Cu(I) binding triggers a marked structural rearrangement in the hCtr1 C-terminal region. The observed Cu(I)-induced conformational remodeling suggests that the C-terminal region may play a dual role, serving both as a channel gate and as a shuttle mediating the delivery of copper ions from the extracellular hCtr1 selectivity filter to intracellular metallochaperones. Our findings thus contribute to a more complete understanding of the mechanism of hCtr1-mediated Cu(I) uptake and provide a conceptual basis for developing mechanism-based therapeutics for treating pathological conditions linked to de-regulated copper metabolism.

INTRODUCTION

All human cells require copper for their metabolic needs. Perturbed copper levels are linked to cancer onset, neurological disorders, metabolic diseases, and anemia (1,2). As such, precise mechanisms regulate the uptake, distribution, storage, and secretion of copper. In the human body, diet-derived copper accumulates in the blood in the oxidation form of Cu(II). Once ingested, Cu(II) is taken up from the blood by the copper transporter hCtr1. The oxidized Cu(II) form is then reduced to the Cu(I) form via an as-yet unclear mechanism.

Ctr1, first identified by Zhou and Gitschier in 1997 (3), is an integral trimeric membrane protein that presents high affinity toward copper (4,5). The human (h)Ctr1 monomer contains 190 amino acids organized such that 60 amino acids assemble into the extracellular N-terminal domain, three transmembrane (TM) helices (TM1, 2, and 3) form a pore, with an intracellular loop of 46 amino acids connecting TM1 and TM2, and finally 15 amino acids are found in a short intracellular C-terminal domain (Fig. 1 *A*). In 2006, Unger et al. reported the first three-dimensional structure of hCtr1 using cryogenic electron microscopy at 6 Å resolution, demonstrating a cone-shaped trimer surrounding a central pore (6). At its narrow end, exposed to the extracellular milieu, the pore diameter is about 0.8 nm and widens to 2.2 nm at the point where copper exits into the cytoplasm.

The TM domain of hCtr1 is characterized by conserved ^{150}MxxxM and ^{167}GxxxG motifs. De Feo and co-workers identified Met150 and Met154 in TM2 as Cu(I)-binding residues, whereas Gly167 and Gly171, both located in TM3, were proposed to mediate a tight interface between TM1 and TM3 (7,8). Schushan and co-workers constructed a Cα-trace model of the TM domain of hCtr1, which agreed well with the experimental structure (9). Based on this model, these authors proposed a transport mechanism in

Submitted August 31, 2021, and accepted for publication February 17, 2022.

[3]These authors contributed equally

*Correspondence: Sharon.ruthstein@biu.ac.il or alessandra.magistrato@sissa.it

Editor: Sudha Chakrapani.
https://doi.org/10.1016/j.bpj.2022.02.033

© 2022 Biophysical Society.
This is an open access article under the CC BY license (http://creativecommons.org/licenses/by/4.0/).

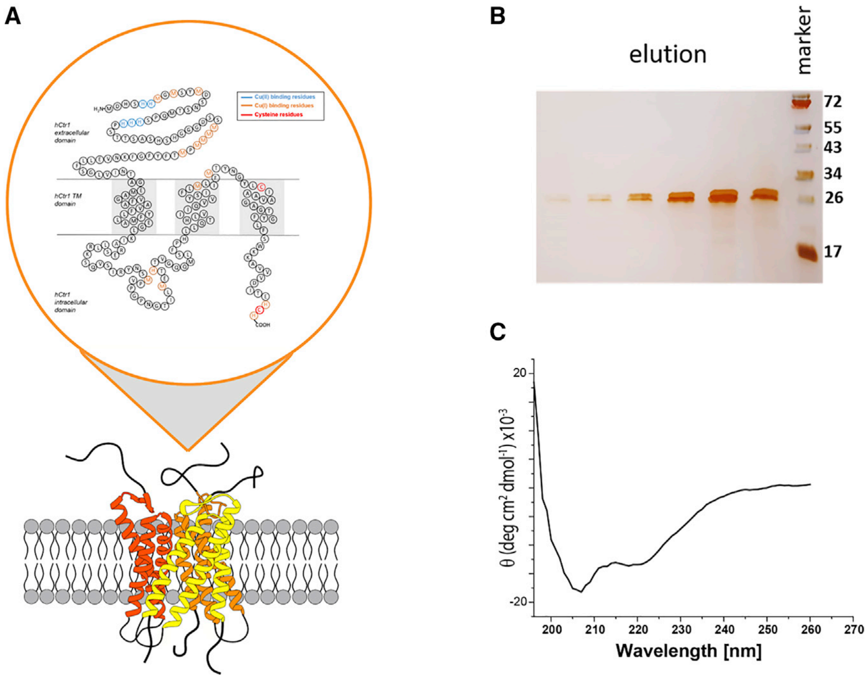

FIGURE 1 Purified hCtr1 characterization. (*A*) Sketch of a membrane-bound hCtr1 trimer. The inset presents the proposed distribution of amino acids within the extracellular, transmembrane (TM), and intracellular domains of the monomer. Residues suggested to be involved in Cu(II) and Cu(I) binding are marked by blue and orange, respectively. Cys residues are marked in red. (*B*) SDS-PAGE of purified hCtr1, after silver staining. (*C*) Circular dichroism analysis of the purified hCtr1.

which Cu(I) ions are transferred one at a time, with Met154, which points to the extracellular exit, along with the conserved His139 and Glu84 residues, located in TM2 and TM1, respectively, controlling transporter motion as a function of metal ion binding and pH. This model of transport is supported by biochemical experiments (5,10) and by the recently reported 3 Å-resolution crystal structure of Ctr1 from *Salmo salar* in the apo and holo forms (i.e., in the absence and presence of Cu(I), respectively) (11). In the holo form, two Cu(I) ions were trapped in the Ctr1 lumen, coordinated by two Met-based triads that included Met146 and Met150 (analogues of Met150 and Met154 in the human variant), respectively. Additionally, residues analogous to His139 and Glu84 were identified as putative Zn(II)-binding sites.

Despite significant advances in unraveling the hCtr1-mediated mechanism of cellular Cu(I) uptake, much remains to be learned. In particular, roles played by the extracellular N-terminal and intracellular C-terminal domains are not yet defined, owing to their disordered nature and hence the absence of a complete high-resolution three-dimensional hCtr1 structure and incomplete biochemical and biophysical characterizations primarily conducted with short model peptides derived from these N- and C-terminal segments.

The extracellular N-terminal domain of hCtr1 serves several roles. These include acquiring copper in the oxidation state of Cu(II) from blood carrier proteins (12), reducing Cu(II) via a still-unknown mechanism to Cu(I) and transferring the Cu(I) to the TM domain. Accordingly, the extracellular N-terminal domain is characterized by two N-glycosylation sites, His-rich sites, and Met-based motifs. Two His-rich sites (i.e., ^{1}MDHxHH and ^{22}HHH) in the extracellular domain have been suggested to bind Cu(II) ions (13,14). These are reduced to Cu(I) via an undefined mechanism and subsequently bind to the Met-rich motifs (^{7}MxMxxM and ^{41}MMMxM) in the extracellular domain with picomolar affinity (15,16). It was shown that ^{41}MMMxM is particularly essential for hCtr1 recruitment of Cu(I) (17,18). However, how Cu(I) ions pass from the last Met-based motif in the extracellular domain to the selectivity filter in the TM domain remains unclear.

The intracellular domain of hCtr1 comprises two parts, namely an intracellular loop between TM1 and TM2, and a short C-terminal tail, which was not resolved in the crystal structure. The C-terminal domain was observed to be somewhat more disordered in the holo than in the apo form of Ctr1 (11), leading to the proposition that conformational changes in the C-terminal tails of the three hCtr1 monomers induced by the presence/absence of Cu(I) modulates metal delivery to various cytoplasmic metallo-chaperones via a cascade of intra-protein or protein-protein interactions (19–21). This proposal is supported by NMR experiments, which revealed that the C-terminal can bind Cu(I) through the terminally located ^{188}HCH motif with high affinity (K_D of 10^{-14} M), and interact with the metallochaperone Atox1 (21). Conversely, mutagenesis experiments showed that the C-terminal ^{188}HCH motif is not crucial for intracellular Cu(I) uptake, complicating the picture (5,10).

To address several of these unresolved issues, we performed electron paramagnetic resonance (EPR) and UV-visible (UV-vis) spectroscopy measurements on the

full-length hCtr1 protein to decipher how Cu(II) and Cu(I) ions bind to hCtr1. Moreover, together with site-directed labeling, EPR distance measurements, and multi-scale all-atom molecular dynamics (MD) simulations, we studied the effect of Cu(I) binding on the conformation of the hCtr1 C-terminal tail. Our results reveal a highly dynamic C-terminal domain, which, depending on Cu(I) concentration, can switch between a pore-sequestered or cytosol-exposed conformation, thus potentially delivering Cu(I) from the channel selectivity filter to metallochaperone in the cytosol for subsequent transfer of the metal (19,21).

MATERIALS AND METHODS

Materials

All chemicals were of high-quality grade. Copper(II) chloride ($CuCl_2 \cdot 2H_2O$), tetrakis(acetonitrile)copper(I) hexafluorophosphate $[Cu(MeCN)_4]PF_6$, bicinchoninic acid (BCA), dithiothreitol (DTT), potassium dihydrogen phosphate (KH_2PO_4), potassium hydrogen phosphate (K_2HPO_4), and HEPES were purchased from Sigma-Aldrich. The solvents acetonitrile and dimethyl sulfoxide (DMSO) were purchased from Bio-Lab. The EPR spin label S-(2, 2, 5, 5-tetramethyl-2, 5-dihydro-1H-pyrrol-3-yl) methyl methanesulfonothioate (MTSSL) was purchased from TRC.

Stock solutions

A 1 M stock solution of DTT was prepared. To make the MTSSL stock solution, 2 mg of MTSSL were dissolved in 120 μL of DMSO to achieve a final concentration of 63 mM. A stock solution of $CuCl_2$ (50 mM) was prepared in distilled water and the concentration was verified by UV-vis spectroscopy from the d-d band of Cu(II) at 780 nm ($\varepsilon = 12\ M^{-1}\ cm^{-1}$). A stock solution (25 mM) of $[Cu(MeCN)_4]PF_6$was prepared in deoxygenated acetonitrile and stored at −20°C until used. EPR ensured that Cu(I) ions had not oxidized. All the stocks were used after further dilution, when needed. The concentration of hCtr1 (molecular weight = 21,090.63; $\varepsilon_{276\ nm}$ = 17,545 $M^{-1}cm^{-1}$) was measured using a NanoDrop microvolume spectrophotometer (Thermo Fisher Scientific).

Experimental methods

Cloning, expression, and purification

Constructs for the expression of wild-type and mutant hCtr1 were prepared by PCR amplification and ligated into a modified pFastBac (pK503-9) insect cell expression vector encoding an N-terminal FLAG tag. Point mutations were PCR amplified using restriction-free (RF) cloning (22). To produce baculovirus for hCtr1 expression, recombinant bacmid was extracted and transfected into Sf9 cells using Cellfectin II Reagent (Thermo Fisher) according to procedures described in the Bac-to-Bac instruction manual (Invitrogen). Insect Sf9 cells were grown at 27°C in protein-free ESF 921 insect cell culture medium (expression systems) in roller bottles and incubated for 3 days post infection. The cells were harvested and re-suspended in 400 mM NaCl, 10% glycerol, 20 mM HEPES buffer, pH = 7.4, lysed, and centrifuged at 40 rpm for 40 min. The pellet was re-suspended in 1.5% Triton X-100, 200 mM NaCl, 10% glycerol, 20 mM HEPES buffer, pH 7.4, and incubated overnight at 4°C. The suspension was centrifuged again at 40 rpm for 40 min. $CaCl_2$ (3 mM) was added to the supernatant, which was loaded onto an anti-FLAG M1 agarose affinity gel (Sigma) column pre-equilibrated with Tris-buffered saline (TBS) buffer (150 mM NaCl, 50 mM Tris-HCl, pH 7.4) and incubated overnight at 4°C. The column was washed with TBS buffer, and the protein was eluted with elution buffer (20 mM HEPES buffer, pH 7.4, 0.1% Triton, 10% glycerol, and 5 mM EDTA). The protein-containing fractions in detergent micelles were collected and analyzed by sodium dodecylsulfate polyacrylamide gel electrophoresis (SDS-PAGE) (14% glycine) and silver staining. Owing to the low yield of the protein, gel filtration was not performed.

Western blot

Aliquots of column samples separated by SDS-PAGE were transferred to a polyvinylidene difluoride membrane using a transfer apparatus according to the manufacturer's protocol (Bio-Rad). After incubation with 3% BSA in TBST (0.5% Tween 20, 150 mM NaCl, 10 mM Tris-HCl, pH 8.0) for 60 min, the membrane was incubated with antibodies against the FLAG tag (1:1000) at 4°C overnight. The membrane was washed with TBST three times for 10 min each time and incubated with a 1:20,000 dilution of peroxidase-conjugated anti-rabbit antibodies for 60 min. The blot was again washed with TBST three times for 10 min each time and developed with an enhanced chemiluminescence system (Bio-Rad) according to the manufacturer's protocol.

Native-PAGE

The purified hCtr1 (pI 6.8) and custom BSA (pI 5) protein (as a marker) in sample buffer containing 250 mM Tris pH 8.5, 30% glycerol, and 0.2 mg/mL bromophenol blue, were analyzed by native-PAGE (12% glycine pH 8.5).

Spin labeling of hCtr1

Wild-type hCtr1 protein was spin labeled with MTSSL. Initially, 2 μL of DTT were added to the protein sample (500 μL), followed by overnight incubation at 4°C with vigorous shaking. The next day, excess DTT was removed using dialysis cassettes (3-kDa cutoff; Pierce). The sample was sonicated for 10 s before spin labeling. Then 1 μL of MTSSL spin label was added to the protein sample, followed by overnight incubation at 4°C with vigorous shaking. Free spin label was removed by several cycles of dialysis over 6 days. The final concentration of the protein was measured using a NanoDrop microvolume spectrophotometer.

X-band continuous wave EPR measurements

Cu(II) low-temperature continuous wave (CW)-EPR measurements were performed using an E500 Elexsys Bruker spectrometer operating at 9.0–9.5 GHz, equipped with a high-sensitivity CW resonator. Spectra were recorded at low temperature (130 ± 5 K) at a microwave power of 20.0 mW, modulation amplitude of 4.0 G, a time constant of 120 ms, and receiver gain of 60.0 dB. The samples were measured in a 1.0-mm quartz tube (Wilmad-LabGlass, Vineland, NJ) placed in a 4.0-mm quartz tube for cooling. CW-EPR simulations were carried out using MATLAB, with the EasySpin toolbox (23).

Q-band double electron-electron resonance experiments

Double electron-electron resonance (DEER) experiments ($\pi/2(\nu_{obs}) - \tau1 - \pi(\nu_{obs}) - t' - \pi(\nu_{pump}) - (\tau1 + \tau2 - t') - \pi(\nu_{obs}) - \tau2$ – echo) were carried out at 50 ± 1.0 K on a Q-band Elexsys E580 spectrometer (equipped with a 2-mm probe head). A two-step phase cycle was employed on the first pulse. The echo was measured as a function of t′, whereas τ2 was kept constant to eliminate relaxation effects. The durations of the observer π/2 and π pulses were 12 and 24 ns, respectively. The duration of the π pump pulse was 24 ns, and the dwell time was 16 ns. τ1 was set to 200 ns. The observer frequency was 33.73 GHz, pump frequency was 33.79 GHz, and the magnetic field was 12,010 G. The samples were measured in 1.6-mm capillary

quartz tubes (Wilmad-LabGlass). The data were analyzed using the DeerAnalysis 2019 program (24). Both Tikhonov regularization (the regularization parameter was carefully chosen based on the best fit of the time domain and the L-curve) and DeerNet were used to analyze the data (25).

UV-vis experiment

UV-vis spectroscopic measurements were performed using a Carry 5000 spectrometer at room temperature (RT). Experiments were carried out using a high-precision cell (purchased from Hellma Analytics) with a 10-mm optical path length cell. The experiments were carried out after baseline corrections of the buffer, since Triton X-100 absorbed in the UV range between 220 and 240 nm. For titration of hCtr1 versus aqueous Cu(II) solution, $CuCl_2$ was used as Cu(II) ions and the stock solution were prepared in double-distilled water. hCtr1 protein (30 μM) was prepared in 20 mM HEPES buffer, pH 7.4, and subsequently titrated with Cu(II) solution (5, 10, 15, 20, 30, 40, 50, 60, 70, 80, and 90 μM). Cu(II) (0.5 or 1 μL) from a 1 mM stock solution was added to the protein-containing mixtures. The total volume of the samples was 100 μL. For titration of hctr1 versus Cu(I) solution, $[Cu(MeCN)_4]PF_6$ was used as the source of Cu(I) ions, dissolved in deoxygenated acetonitrile and 20 mM HEPES buffer, pH 7.4. hCtr1 protein (20 μM) was prepared in 20 mM HEPES buffer, pH 7.4, and then titrated with Cu(I) solution (5, 10, 15, 20, 30, 40, 50, 60, 70, 80, 90, 100, 110, 120, 130, 140, 150, 160, 170, 180, 190, and 200 μM). Cu(I) (0.5 or 1 μL) from a 1 mM stock was added to the protein-containing mixtures.

Circular dichroism characterization

Circular dichroism (CD) measurements were performed using a Chirascan spectrometer (Applied Photophysics, UK) at RT. Measurements were carried out in a cell with 1-mm optical path length. The data were recorded from 190 to 260 nm with a step size and a bandwidth of 1 nm. Spectra were obtained after background subtraction. The data were analyzed with the CDNN program.

Computational methods

System setup

The atomic structure of hCtr1 was generated using MODELLER (version 10.0) homology modeling software (26). The crystal structure of Ctr1 from *S. salar* (PDB: 6m97) (11) served as template. Out of 10 generated structures, the model with the lowest Discrete Optimized Protein Energy (DOPE) score was selected for loop refinement (residues 97–128, 288–315, and 478–504). The final structure was anchored in a 1-Palmitoyl-2-oleoylphosphatidylcholine (POPC) membrane bilayer and solvated with TIP3P water using the CHARMM-GUI Membrane Builder (27). The simulation box was 110 Å × 110 Å × 120 Å in size. Na^+ and Cl^- were added to reach a final ionic concentration of 0.15 M. Forces in the system were described using Amber force fields ff14SB (28) (protein) and lipid17 (29) (membrane). Cu(I) sites were described using a bonded model. Two Cu(I) ions were bound to the selectivity filter composed of Met150 and Met154. One Cu(I) was bound to each ^{188}HCH motif. Metal Center Parameter Builder (MCPB) (30) and Gaussian09 (31) were used to optimize the binding sites and extract relevant force-field parameters. Calculations were performed with the Becke, 3-parameter, Lee-Yang-Parr (B3LYP) (32,33) functional and 6-31G* basis set (34).

QM/MM MD simulations

Binding of Cu(I) to the selectivity filter and C-terminal end of hCtr1 was predicted with a hybrid quantum mechanics (QM)-molecular mechanics (MM) MD simulation. For the C-terminally located Cu(I)-binding site, calculations were performed using a short model peptide, corresponding to the last nine amino acids of hCtr1. The peptide was solvated in TIP3P water (box size 37 Å × 41 Å X 35 Å) and Cl^- ions were added to achieve neutrality. A 100-ns classical MD (protocol described below) was run to relax the peptide around the Cu(I)-bound ^{188}HCH motif. Next, QM-MM MD simulations were performed with cp2k (version 7.1) (35). To pinpoint the coordination geometry of Cu(I) ions binding in the selectivity filter, two copper ions, and the side chains of Met150 and Met154 were included in the QM box, while in simulations focused on Cu(I) binding to the C-terminal peptide, the quantum portion was composed of Cu(I) and the side chains of residues His188, Cys189, and His190. In all cases, the QM/MM boundary was across the Cβ–Cα bond. The quantum portion was described with the Becke-Lee-Yang-Parr density functional theory (DFT/BLYP) (33,36) using the double-ζ molecularly optimized basis set along with Goedecker–Teter–Hutter pseudopotentials (DZVP-MOLOPT-GTH) (37). For the copper ion, the short range version of the basis set was used (DZVP-MOLOPT-SR-GTH). The system was initially minimized in the microcanonical (NVE) ensemble and slowly heated to 300 K using the canonical (NVT) ensemble. After equilibration, the trajectory was followed for 2.5 ps. Binding sites were re-parametrized based on the final geometry using MCPB (30) and Gaussian09 (31) to derive Cu-site-specific force-field parameters. Force constants and Merz-Kollman restrained electrostatic potential (RESP) charges were obtained with the B3LYP functional and 6-31G* basis set. Modified force-field parameters were used in all subsequent classical MD runs.

Classical MD simulations

Classical MD simulations were performed using the GROMACS software package (version 2020.3) (38). Simulated systems were first relaxed in an NVT ensemble for 10 ns using periodic boundary conditions and position restraints on the protein component. To prevent the membrane from splitting open during the equilibration of hCtr1, a position restraint in the z direction perpendicular to the membrane was placed on atom P31 in the lipid residues. Equilibration was continued for another 10 ns in the isothermal–isobaric (NPT) ensemble to stabilize the pressure, followed by a 100-ns production phase. Newton's equations of motion were integrated using the leap-frog algorithm and a 2-fs time step. Electrostatic interactions were evaluated using the particle mesh Ewald method. Temperature was noted using a Nose-Hoover thermostat (coupling constant was 0.5 ps), while a Parrinello-Rahman barostat was used for pressure control ($\tau_p = 2$). All restraints were removed during the production run. Trajectories were analyzed using Python package MDTraj (39).

RESULTS

This study aims to obtain biophysical properties of the hCtr1 membrane protein. hCtr1 was purified from insect cells using anti-FLAG M1 beads (Fig. 1 *B*). The eluted fractions were examined by western blot using anti-Ctr1 antibodies and by native gel (Fig. S1), which confirmed the trimerization of the hCtr1 protein. We additionally performed CD spectroscopy of the purified protein to determine the secondary structure and folding properties of the protein. The CD spectrum (Fig. 1 *C*) shows the dominant presence of α helices (90% ± 3%) in the secondary structure. Next, we characterized the Cu(II) and Cu(I) binding to hCtr1, and the conformational changes in the C-terminal tail induced by Cu(I).

Cu(II) coordination to hCtr1

The extracellular domain of hCtr1 contains the His-rich sites ^{1}MDHxHH and ^{22}HHH, which were suggested to serve as

Cu(II)-binding sites (13,14). To evaluate the amount of Cu(II) ions that can bind to hCtr1, low-temperature (130 K) CW-EPR and RT UV-vis experiments were conducted (Fig. 2). The EPR data were analyzed using EasySpin and the g- and the A-tensor were determined (Table 1). At a ratio of 0.5:1 Cu(II):hCtr1, the signal was characterized by two components. The dominant species (accounting for 75%), with a two- or three-nitrogen coordination environment (2N2O/3N1O), based on the g- and the A-tensors (40), and 25% with either three or four oxygen coordination environment (1N3O/4O). Between 0.8 and 2 Cu(II):hCtr1, the EPR spectrum was characterized by a single component, with 2N2O/3N1O coordination. Three pulsed electron spin echo enveloped modulation (3P-ESEEM) carried out on 1:1 Cu(II):hCtr1 (Fig. S1) confirmed that Cu(II) is coordinated to nearby ^{14}N nuclei. At a higher ratio, the EPR spectrum is characterized again by two components, where the second component was characterized by three to four oxygen coordination. The ratio of the second component (1N3O/4O) increases with increasing Cu(II) concentration, suggesting that this component is a hydrated Cu(II) ion. Overall, the CW-EPR spectrum suggests that hCtr1 monomer can bind up to two Cu(II) ions.

UV-vis spectroscopy experiments were performed to evaluate the number of Cu(II) ions bound to a hCtr1 monomer (Fig. 2 *B*). The resulting absorption spectrum shows a maximum at 276 nm, which corresponds to the absorption of aromatic amino acid residues (Trp, Tyr, and Phe) present in hCtr1. The addition of Cu(II) to the protein solution increased the intensity of the absorption maximum up to a 2:1 ratio of Cu(II)/hCtr1, most likely owing to binding to residues near the aromatic residues. The UV-vis experiments confirmed the EPR data showing that at least two Cu(II) ions can coordinate to one hCtr1 monomer.

Cu(I) coordination to hCtr1

UV-vis experiments were also conducted to evaluate the number of bound Cu(I) ions per hCtr1 monomer. To this end, Cu(I) ions were added to hCtr1 solution, and subsequent changes in the absorption spectrum of hCtr1 were monitored by UV-vis spectroscopy (Fig. 3 *A*). The absorption spectrum of hCtr1 shows a maximum at 276 nm, whose intensity increases with the progressive addition of Cu(I) similarly to what was observed upon Cu(II) binding. However, Cu(I) addition to hCtr1 solution shifted the absorption peak of hCtr1 from 276 nm to 265 nm, whereas adding higher Cu(I) amounts, above 3.5 equivalents, resulted in a shift to 270 nm. The blue shift from 276 nm to 265 nm corresponds to the Cu(I) to sulfur atoms charge transfer effect, suggesting the involvement of Met residues in Cu(I) binding (41). At high Cu(I) concentrations above 3.5 equivalent of Cu(I), other amino acids (beside Cys or Met) assume roles in Cu(I) coordination, and a red shift from 265 nm to 270 nm was detected.

In addition, to determine the binding ratio of Cu(I) to hCtr1, we plotted the change in the absorption intensity of hCtr1 at 270 nm (similar results would be obtained at 276 nm or 265 nm) upon Cu(I) titration versus the ratio between the concentrations of Cu(I) and hCtr1. We noted that the intensity of the peak at 270 nm sharply increased until the addition of five equivalents of Cu(I), suggesting that five Cu(I) ions bind to each hCtr1 monomer. This is similar to a value obtained in earlier studies, where it was shown that three to four Cu(I) ions can bind to the extracellular

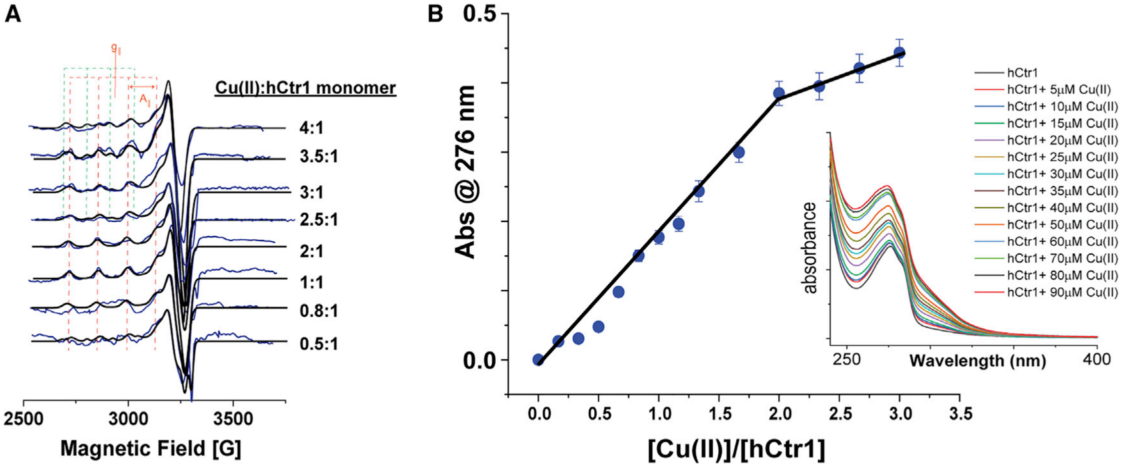

FIGURE 2 Cu(II) coordination to hCtr1. (*A*) CW-EPR spectra (acquired at 130 K) for various Cu(II) concentrations in the presence of 80 μM hCtr1 monomer in 20 mM HEPES buffer with 0.1% Triton, pH 7.4. The EPR spectra are shown as blue solid lines, while dark solid lines represent simulated data. Parameters of the simulations are given in Table 1. The orange and green dashed lines represent the g_{II} and A_{II}values of the two components. (*B*) Plots of absorbance at 276 nm versus Cu(II):hCtr1 ratio ([hCtr1] = 30μM) from the corresponding UV-vis spectra (inset) indicate two equivalents of Cu(II) bind to hCtr1 monomer. The error bars were evaluated using the difference in absorption between 275 and 277 nm.

TABLE 1 EPR parameters for Cu(II) bound to hCtr1 obtained from EasySpin simulations

Cu(II):hCtr1	g-tensor (±0.0005)	A-tensor [MHz] (±5 MHz)	% Species (±5%)	Coordination
0.5:1	[2.07 2.28]	[20,460]	75	2N2O/3N1O
	[2.03 2.34]	[20,420]	25	4O/1N3O
0.8:1	[2.06 2.28]	[20,470]	100	2N2O/3N1O
1:1	[2.06 2.28]	[20,470]	100	2N2O/3N1O
2:1	[2.06 2.28]	[20,470]	100	2N2O/3N1O
2.5:1	[2.06 2.28]	[20,470]	90	2N2O/3N1O
	[2.055 2.34]	[25,390]	10	4O/1N3O
3:1	[2.06 2.28]	[20,470]	80	2N2O/3N1O
	[2.055 2.34]	[25,390]	20	4O/1N3O
3.5:1	[2.06 2.28]	[20,470]	70	2N2O/3N1O
	[2.055 2.34]	[25,390]	30	4O/1N3O
4:1	[2.06 2.28]	[20,470]	50	2N2O/3N1O
	[2.055 2.34]	[25,390]	50	4O/1N3O

Simulations were made using the Pepper program (23).

N-terminal domain (16,42), one Cu(I) ion binds to the C-terminal tail (21), and two Cu(I) ions are found in the selectivity filter (11). Cu(I) additions above five equivalents slowed the intensity increase at 270 nm, although no plateau was reached. This may be due to either Cu(I) exchange over several binding sites within a hCtr1 monomer or Cu(I) saturation/precipitation in solution (43,44).

Next, to ascertain that each hCtr1 monomer can bind up to five Cu(I) ions, we employed bicinchoninic acid, a high-affinity Cu(I) chelator (45) (Fig. 3 *B*). Two molecules of BCA bind a Cu(I) ion to form a $(BCA)_2$-Cu(I) complex, a chromophore that shows high-intensity peaks at 562 nm and 354.5 nm. Initially, we formed a 25 μM $(BCA)_2$-Cu(I) complex by mixing 60 μM BCA, 25 μM Cu(I), and a small excess of BCA ligand to ensure that no free Cu(I) was available in the solution. Then, $(BCA)_2$-Cu(I) was titrated upon addition of hCtr1 protein. The UV-vis spectrum of the BCA ligand did not show any absorption in the visible region. The addition of 25 μM Cu(I) into 60 μM BCA solution resulted in an intense peak at 562 nm, confirming formation of the $(BCA)_2$-Cu(I) complex. Subsequent addition of hCtr1 in $(BCA)_2$-Cu(I) solution decreased the intensity of the absorption band at 562 nm. The addition of 5 μM hCtr1 completely eliminated absorption at 562 nm, which suggests removal of Cu(I) from BCA coordination. Similarly, the intensity of the absorption band at 354.5 nm decreased with the addition of hCtr1 and also restored the absorption of BCA ligand at 335 nm (a peak at 335 nm was found to be BCA concentration dependent) (Fig. S2). When we carried out a UV-vis experiment in which the hCtr1-Cu(I) complex was established with a stoichiometric ratio of 1:5 and then titrated with BCA ligand, no peak at 562 nm for $(BCA)_2$-Cu(I) was observed, although an increase in the BCA concentration-dependent peak at 335 nm was seen (Fig. S3) Thus, the UV-vis data confirm that hCtr1 has a stronger Cu(I)-binding affinity than does the BCA ligand. Finally, 5 μM hCtr1 removed 25 μM Cu(I) from 25 μM $(BCA)_2$-Cu(I), confirming that a hCtr1 monomer can bind five equivalents of Cu(I).

Conformational changes in the C-terminal tail of hCtr1 upon Cu(I) binding

EPR spectroscopy with site-directed spin labeling can provide information on the dynamics and conformational changes of protein side chains and has become widely used in biophysical research (46–52). An electron spin introduced into a diamagnetic protein provides information on the local environment of the spin label and on the mobility of the protein domain (53–56). The spin label, commonly attached to cysteine residues, is the methanesulfonothioate (MTSSL) nitroxide radical (Scheme S1). In hCtr1, there are two Cys residues to which the spin label may attach. Cys161, located in the transmembrane domain, might not be available for spin labeling owing to its location in the channel lumen, whereas Cys189 is found in the C-terminal intracellular region of the protein (Fig. 1 *A*). Initially, we carried out RT CW-EPR experiments to detect whether there is a change in the dynamics of spin-labeled hCtr1 protein upon Cu(I) binding (Fig. S4). The EPR spectra in the absence or presence of Cu(I) were similar and were characterized by two species (in a 1:1 ratio). One species had high dynamics with a correlation time of 1×10^{-10} s and an electron-electron interaction of 6.0 MHz (corresponding to an average distance of 2.0 nm). The other species was simulated with correlation time of 1×10^{-7} s and showed no electron-electron interactions. Field sweep pulsed EPR experiments and two-pulse echo decay signals (Fig. S5) did not detect changes as a function of Cu(I) coordination, confirming that the presence of Cu(I) did not affect the assembly of the Ctr1 membrane protein (57,58).

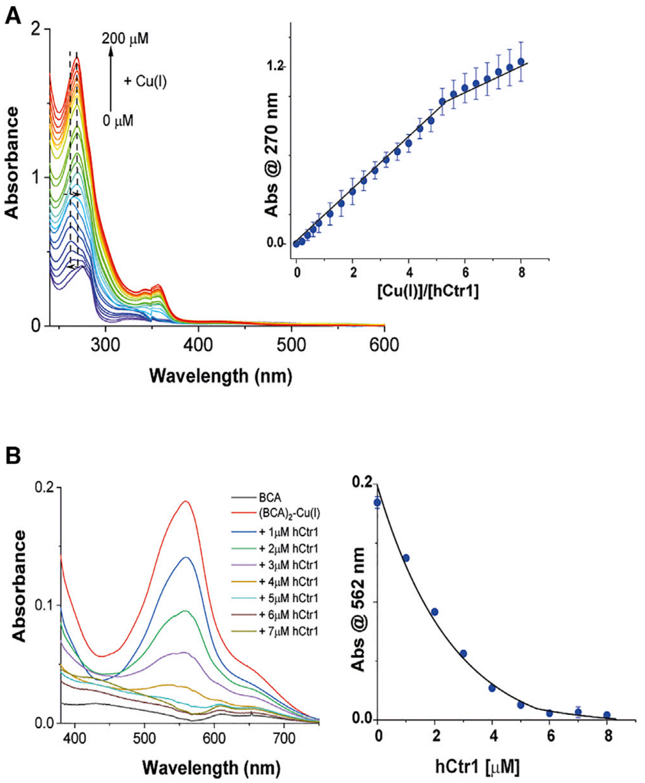

FIGURE 3 Cu(I) coordination to hCtr1. (*A*) The UV-vis spectra of the titration of Cu(I) to hCtr1 solution. Inset shows the plot of absorbance at 270 nm versus [Cu(II)]/[hCtr1] showing that five equivalents of Cu(I) bind to a hCtr1 monomer. The absorbance at 270 nm of hCtr1 alone was subtracted from respective absorbance values obtained for hCtr1 alone and hCtr1 after addition of Cu(I). The error bars were evaluated using the difference in absorption between 271 and 269 nm. (*B*) The UV-vis spectra of the titration of hCtr1 to $(BCA)_2$-Cu(I) solution (left) and decrease in intensity at 562 nm upon hCtr1 addition (right). The concentrations of BCA and Cu(I) were 60 μM and 25 μM, respectively, to form a 25 μM $(BCA)_2$-Cu(I) complex. Measurements were carried out in 20 mM HEPES buffer with 0.1% Triton, pH 7.4.

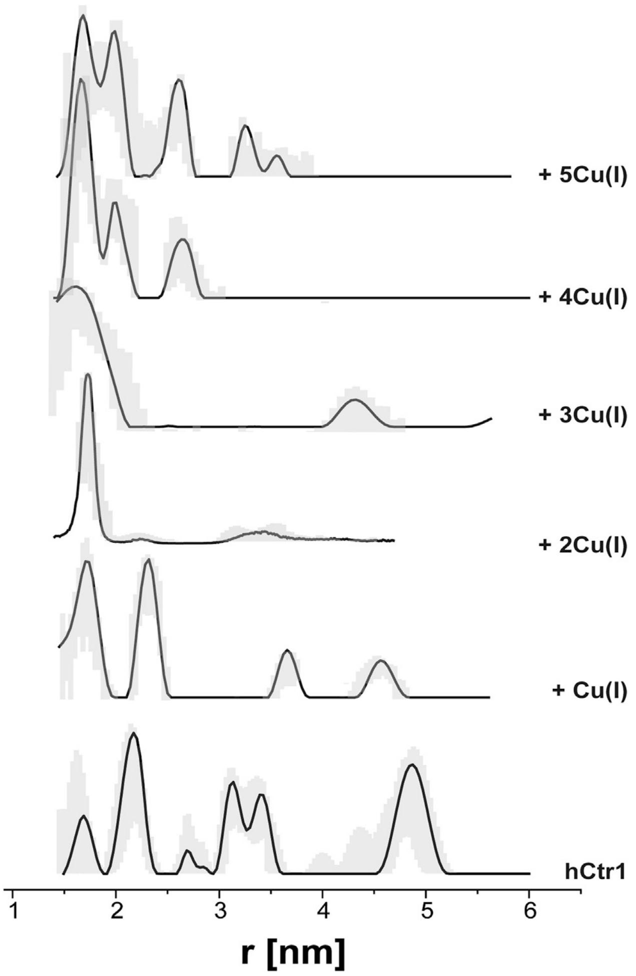

FIGURE 4 Q-band DEER distance distribution functions of spin-labeled wild-type hCtr1 protein in the absence or presence of Cu(I) in 20 mM HEPES buffer with 0.1% Triton, pH 7.4. The gray line corresponds to the uncertainty.

We next performed EPR distance distribution experiments, named DEER, to evaluate distances between the spin-labeled Cys residues in hCtr1 protein as a function of Cu(I) concentration (Figs. 4 and S6). In the absence of Cu(I), several peaks were observed in the DEER spectrum, spanning the range from 1.5 to 5.5 nm. The data were analyzed using both Tikhonov regularization as well as DeerNet (25) (Fig. S7). Taking into account the uncertainty in the analysis of both methods, the following distributions exists for apo-hCtr1: 2.1 ± 0.3 nm, and 3.3 ± 0.3 nm. Moreover, the analysis also suggests a distribution of around 5.0 nm; however, owing to the short-time-domain DEER signal, this distribution cannot be accurately determined. The addition of Cu(I) ions triggered a decrease in the number of observed peaks in the distance distribution, suggesting that hCtr1 assumed a more rigid and symmetric structure. Intriguingly, at a two Cu(I):one hCtr1 monomer ratio, we observed a single narrow peak around 1.7 ± 0.1 nm, and at a ratio of a three Cu(I):one hCtr1 monomer ratio, a distribution of 1.6 ± 0.3 nm is detected. Further increasing Cu(I) concentration induced appearance of additional peaks above 2.0 nm, signaling re-acquisition of a flexible structure.

To assess the spin labeling of Cys161, we expressed and performed DEER experiments on the hCtr1_C161A mutant. Comparing the distance distribution function of wild-type hCtr1 and the hCtr1_C161A mutant at various Cu(I) concentrations (Fig. S8) suggests that Cys161 was at least partially spin labeled.

To examine the effect of Cu(I) binding on hCtr1 conformation in detail, we performed all-atom MD simulations. First, homology modeling based on the crystal structure of Ctr1 from *S. salar* (11) was performed to obtain the atomic-level structure of hCtr1 (Fig. 5 *A*). The intrinsically disordered 44-amino-acid-long N-terminal end was not included in the model due to the lack of structural data. Next, Cu(I) was added to the structure. Two Cu(I) ions

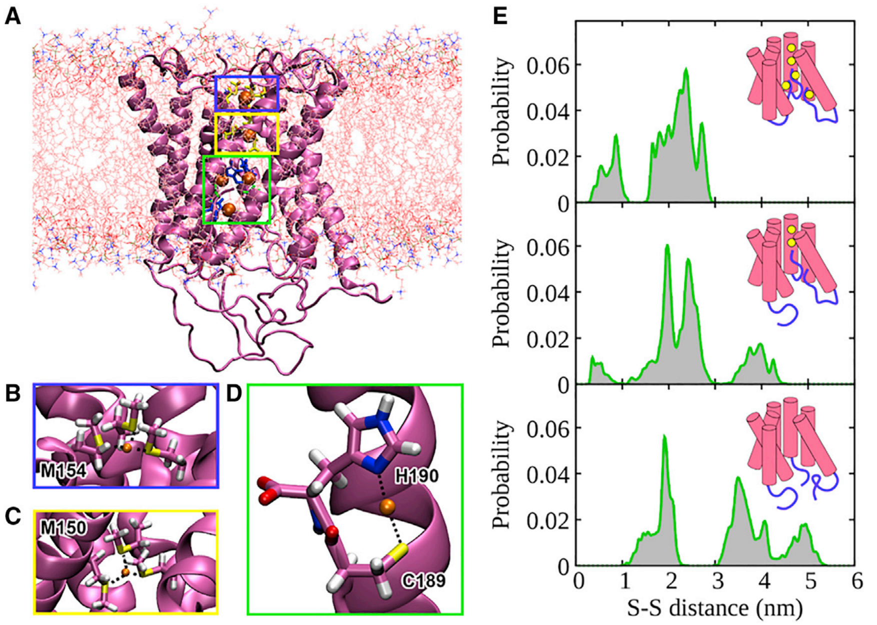

FIGURE 5 Molecular dynamics (MD) simulations of hCtr1. (*A*) Model of hCtr1 lacking the intrinsically disordered N-terminal end embedded in a phosphatidylcholine (POPC) membrane. The protein is depicted in its holo-form (i.e., with two Cu(I) ions bound in the selectivity filter and one ion to each C-terminal region). (*B* and *C*) Cu(I) coordination sphere in the top and bottom Met-based triads, respectively. (*D*) Cu(I) bound to the hCtr1 C-terminal end via Cys189 and His190. The protein is shown as a pink cartoon with atoms defining the binding site shown in licorice, and S, H, N, O, and C depicted in yellow, white, blue, red, and pink, respectively. Cu(I) is represented as an orange van der Waals sphere and its coordination sphere is highlighted with black dashed lines. (E) Distributions of pair distances between Cys sulfur atoms@Cys189 and Cys161 as obtained from MD trajectories. The cartoon shows the hCtr1 model with the transmembrane helices depicted as pink cylinders and each C terminus represented by a violet line. Copper is shown as a yellow circle.

were positioned into the selectivity filter (Figs. 5 *B* and *C*), one into each Met-based triad that included Met150 (bottom triad) and Met154 (top triad), respectively, as observed in the crystal structure. In contrast, there is no structural information available regarding Cu(I) binding to the C-terminal end beyond the involvement of the ^{188}HCH motif. Since classical MD simulations rely on pre-defined empirical force fields to describe the system, they are unable to accurately describe the coordination spheres of metal ions (59). Hence, to predict the coordination geometry of the different Cu(I)-binding sites, 2.5 ps of hybrid DFT-based QM/MM MD simulations were initially performed (see section "materials and methods") (60). When considering the binding of Cu(I) in the selectivity filter, simulations were begun from the position occupied in the crystal structure (PDB: 6m98) (11), where both Cu(I) ions lie below the plane of the Met-triad to which they bind and are 7.22 Å apart. The coordination distance is 2.2 Å for Cu(I)-S@Met in the top triad and 3.2 Å in the bottom triad. After QM/MM MD simulation, the Cu(I) ions were positioned 6.2 ± 0.4 Å apart, with an average coordination distance of 2.3 ± 0.1 Å for Cu(I)-S@Met in both the top and bottom Met-based triads. Only one of the Met residues in the bottom triad exhibited a larger fluctuation within the coordination distance (2.4 ± 0.3 Å) (61). The root-mean-square deviation (RMSD) of the QM/MM MD-optimized selectivity filter was 5.3 Å with respect to the crystal structure (Fig. S9). When considering the binding of Cu(I) to the C-terminally located binding site, QM/MM MD simulation was initiated with the Cu(I) ion coordinated to all three residues in the ^{188}HCH motif. Coordinating atoms were Nε@His188, S@Cys189, and Nε@His190. During the 2.5-ps simulation, His188 rapidly dissociated from Cu(I), while Cys189 and His190 remained bound to copper in a linear spatial arrangement (Fig. 5 *D*). The resulting average distances between Cu(I) and the coordinating atoms were 2.21 ± 0.05 Å for Cu(I)-S@Cys189 and 1.95 ± 0.05 Å for Cu(I)-Nε@His190. After having relaxed each Cu(I) coordination sphere at the QM level, we derived Cu-site-specific force-field parameters and performed classical MD simulations to explore the conformational plasticity of hCtr1 in the presence of an increasing amount of Cu(I). One-hundred-nanosecond MD simulations were performed for (1) apo-hCtr1, (2) hCtr1 with two Cu(I) ions in the selectivity filter, and (3) holo-hCtr1 with one Cu(I) ion bound to each of the three C-terminal ends, in addition to the two Cu(I) ions found in the selectivity filter. Moreover, in each case, several conformations of the C-terminal ends were sampled. Specifically, we considered models differing in the number of C-terminal ends positioned inside or outside the hCtr1 pore. In total, 12 simulations were performed (Fig. S10). From the MD simulation trajectories obtained, we monitored the distributions of pair distances between the sulfur atoms of Cys161 and Cys189 and compared them with the DEER data, where Cys161 and Cys189 were labeled with MTSSL. This allowed us to infer the molecular basis of the changes in distance distributions observed in the DEER spectra as Cu(I) concentration was increased (Fig. 5 *E*). Strikingly, this comparison suggested that, when hCtr1 is fully in the apo-form (i.e., with no bound Cu(I) ions), the C-terminal ends are oriented outward, toward the cytoplasm (Fig. 5 *E*, bottom panel). Upon binding of Cu(I) ions in the selectivity filter, C-terminal tails start to move into the hCtr1 pore, resulting in a decrease of the peak at 5 nm and in the occurrence of a strong peak at 2.5 nm (Fig. 5 *E*, middle panel). The single peak observed

in Q-band DEER experiments at higher Cu(I):hCtr1(monomer) molar ratios (2:1 and 3:1), when all available Cu(I)-binding sites are occupied, only fits a model in which all three C-terminal ends are found within the hCtr1 pore (Fig. 5 *E*, top panel). DEER spectra obtained at higher Cu(I) concentrations matched the distance distributions obtained from MD simulations less well. However, due to the re-occurrence of peaks at distances above 3 nm, we posit the C-terminal tails move back toward the cytoplasm, as suggested by a recent X-ray structure (11). Since experiments were performed on the MTSSL-labeled hCtr1, we even performed the MD simulations in the apo state by also adding the MTSSL label to the protein. Inclusion of the label did result in modest shifting of certain peaks, but, overall, the distance distributions were comparable with those obtained from simulations of non-labeled hCtr1, indicating S-S distance distributions are a good approximation for the distances measured in the DEER experiment (Fig. S11).

DISCUSSION

The homotrimeric structure of hCtr1 encompassing a selectivity filter composed of two sets of Met-based triads, coupled with apparently energy-independent Cu(I) transport across the membrane, suggests that hCtr1 functions like an ion channel (7). Nevertheless, the conduction rate for Cu(I) ions was estimated to be ~10 ions per second per hCtr1 trimer in cell culture, a rate that is uncharacteristically slow for an ion channel (5). It was speculated that the low conduction rate might be due to gating of the channel by the C-terminal domains. The latter may essentially act as a plug, regulating the import of potentially toxic Cu(I) ions from hCtr1 and its transfer to the Atox1 chaperone for subsequent delivery to the different Cu(I) trafficking routes. This concept is supported by the observation that the C-terminal end in the crystal structure of Ctr1 from *S. salar* was slightly better resolved in the apo state, indicating that Cu(I) binding slightly increases Ctr1 conformational flexibility (5,11).

Three additional distinct lines of evidence support the possibility that the Ctr1 C-terminal domain functions as an intracellular gate. First, fluorescence resonance energy transfer (FRET) experiments demonstrated that the C-terminal end moves during copper transport (62). Second, C-terminal truncations of Ctr1 or mutation of the ^{188}HCH motif exhibited substantially elevated transport rates in cell culture (10,17). Third, *in vitro* mutagenesis studies demonstrated that mutation of His139, located in the channel pore close to the exit, to Arg, increased the transport rate fourfold (5,10). While it was initially suggested that this mutation altered the pore shape due to electrostatic repulsion between Arg residues, it might also prevent the intra-pore conformation of the C-terminal tail, either through steric hindrance or due to disruption of stabilizing interactions between the ^{188}HCH motif and His139. The latter hypothesis is supported by the observation that the H139R and ^{188}AAA mutants show no synergistic effect and, therefore, target the same mechanism (5). Nevertheless, the role of the C-terminal region in the Ctr1 mechanism may be diverse, as it has been shown that C-terminal tail deletion abolishes Cu-mediated endocytosis (11).

In this study, we addressed the proposed conformational plasticity of the hCtr1 C-terminal tail and its role in the hCtr1-mediated Cu(I) transport. Our data demonstrate that each hCtr1 monomer binds up to five Cu(I) ions and compellingly support the idea that Cu(I) binding triggers a marked structural rearrangement of the C-terminal region as the number of bound Cu(I) ions increases. By comparing DEER spectra with all-atom MD simulations, we revealed that the C-terminal tails are most likely found in the cytoplasm when hCtr1 is in its fully apo form and move toward the pore when the metals occupy the selectivity filter and the C-terminal end, resulting in a more symmetric structure. Finally, at higher concentrations of Cu(I), as each hCtr1 monomer binds five Cu(I) ions, the tails start to become more flexible again and most likely undergo an outward/inward movement between the hCtr1 extracellular pore region and the cytosol. However, the mechanism that triggers the observed conformational changes remains unclear and will need to be further explored in future studies. Additionally, since the extracellular N-terminal end also binds Cu(I), it might also mediate the conformational change. However, due to its disordered structure and lack of structural detail regarding Cu(I) coordination sites, the role of this domain could not be resolved in the present study. Additionally, while movement of the C-terminal tail has been demonstrated in situ in yeast cells (62), the details of the conformational behavior in the cellular environment might be different, since the DEER experiments were performed in the presence of detergent micelles, which can affect the flexibility and dynamic behavior of the membrane protein (63).

Taken together, our results contribute to defining the role of the Ctr1 C-terminal region in the internalization of Cu(I) ions. A detailed understanding of Cu(I) cell entry may unlock new avenues for developing mechanism-based therapeutics to treat various pathological conditions linked to abnormal copper metabolism (64).

SUPPORTING MATERIAL

Supporting material can be found online at https://doi.org/10.1016/j.bpj.2022.02.033.

AUTHOR CONTRIBUTIONS

G.W., H.K., S.M., and Y.S. performed experimental research and analyzed data. J.A. and P.J. performed computational research. Z.Q. and L.G.-A. assisted in the experimental research. J.A., A.M., G.W., and S.R. designed the research and wrote the paper.

ACKNOWLEDGMENTS

This research was funded by ERC-STG grant no. 754365 awarded to S.R. J.A. kindly acknowledges the Giovanni Fraviga Italian Association for Cancer Research (AIRC) fellowship. P.J. was supported by the HPC Europa Fellowship (HPC17M882U) under the project HPC-EUROPA3 (INFRAIA-2016-1-730897). A.M. thanks the Italian Association for Cancer Research (project AIRC IG 24514) and regional operational program ERDF 2014-2020 of the Friuli Venezia Giulia Region (project ARES - Against bRain cancEr: finding personalized therapies with in Silico and in vitro strategies) for financial support.

REFERENCES

1. Rosenzweig, A. C. 2001. Copper delivery by metallochaperone proteins. *Acc. Chem. Res.* 34:119–128.
2. Uriu-Adams, J. Y., and C. L. Keen. 2005. Copper, oxidative stress, and human health. *Mol. Aspect. Med.* 26:268–298.
3. Zhou, B., and J. Gitschier. 1997. hCTR1: a human gene for copper uptake identified by complementation in yeast. *Proc. Nat. Acad. Sci.* 94:7481–7486.
4. Lee, J., M. M. O. Pena, …, D. J. Thiele. 2002. Biochemical characterization of the human copper transporter Ctr1. *J. Biol. Chem.* 277:4380–4387.
5. Maryon, E. B., S. A. Molloy, …, J. H. Kaplan. 2013. Rate and regulation of copper transport by human copper transporter 1 (hCTR1). *J. Biol. Chem.* 288:18035–18046.
6. Aller, S. G., and V. M. Unger. 2006. Projection structure of the human copper transporter CTR1 at 6A resolution structure reveals a compact trimer with a novel channel-like architecture. *Proc. Nat. Acad. Sci.* 103:3627–3632.
7. De Feo, C. J., S. G. Aller, …, V. M. Unger. 2009. Three-dimensional structure of the human copper transporter hCTR1. *Proc. Nat. Acad. Sci.* 106:4237–4242.
8. De Feo, C. J., S. G. Aller, and V. M. Unger. 2007. A structural perspective on copper uptake in eukaryotes. *Biometals.* 20:705–716. https://doi.org/10.1007/s10534-006-9054-7.
9. Schushan, M., Y. Barkan, …, N. Ben-Tal. 2010. Cα-trace model of the transmembrane domain of human copper transporter 1, motion and functional implications. *Proc. Nat. Acad. Sci.* 107:10908–10913.
10. Eisses, J. F., and J. H. Kaplan. 2005. The mechanism of copper uptake mediated by human CTR1: a mutational analysis. *J. Biol. Chem.* 280:37159–37168.
11. Ren, F., B. L. Logeman, …, P. Yuan. 2019. X-ray structures of the high-affinity copper transporter Ctr1. *Nat. Commun.* 10:1386.
12. Shenberger, Y., A. Shimshi, and S. Ruthstein. 2015. EPR spectroscopy shows that the blood carrier protein, human serum albumin, closely interacts with the N-terminal domain of the copper transporter, Ctr1. *J. Phys. Chem. B.* 119:4824–4830.
13. Shenberger, Y., O. Marciano, …, S. Ruthstein. 2018. Insights into the N-terminal Cu(II) and Cu(I) binding sites of the human copper transporter CTR1. *J. Coord. Chem.* 71:1985–2002.
14. Haas, K. L., A. B. Putterman, …, K. J. Franz. 2011. Model peptides provide new insights into the role of histidine residues as potential ligands in human cellular copper acquisition via Ctr1. *J. Am. Chem. Soc.* 133:4427–4437.
15. Jiang, J., I. A. Nadas, …, K. J. Franz. 2005. A mets motif peptide found in copper transport proteins selectively binds Cu(I) with methionine-only coordination. *Inorg. Chem.* 44:9787–9794.
16. Du, X., H. Li, …, H. Sun. 2013. Kinetics and thermodynamics of metal binding to the N-terminus of a human copper transporter, Hctr1. *Chem. Commun. (Camb).* 49:9134–9136.
17. Larson, C. A., P. L. Adams, …, S. B. Howell. 2010. The role of the N-terminus of mammalian copper transporter 1 in the cellular accumulation of cisplatin. *Biochem. Pharm.* 80:448–454.
18. Puig, S., J. Lee, …, D. J. Thiele. 2002. Biochemical and genetic analyses of yeast and human high affinity copper transporters suggest a conserved mechanism for copper uptake. *J. Biol. Chem.* 277:26021–26030.
19. Levy, A. R., V. Yarmiayev, …, S. Ruthstein. 2014. Probing the structural flexibility of the human copper metallochaperone Atox1 dimer and its interaction with the CTR1 C-terminal domain. *J. Phys. Chem. B.* 118:5832–5842.
20. Shenberger, Y., V. Yarmiayev, and S. Ruthstein. 2013. Exploring the interaction between the human copper transporter, CTR1, C-terminal domain and a methionine motif in the presence of Cu(I) and Ag(I) ions, using EPR spectrosocopy. *Mol. Phys.* 111:2980–2991.
21. Kahra, D., M. Kovermann, and P. Wittung-Stafshede. 2016. The C-terminus of human copper importer Ctr1 acts as a binding site and transferes copper to Atox1. *Biophys. J.* 110:95–102.
22. Unger, T., Y. Jacobovitch, …, Y. Peleg. 2010. Applications of the Restriction Free (RF) cloning procedure for molecular manipulations and protein expression. *J. Struct. Biol.* 172:34–44.
23. Stoll, S., and A. Schweiger. 2006. EasySpin, a comprehensive software package for spectral simulation and analysis in EPR. *J. Magn. Reson.* 178:42–55.
24. Jeschke, G. 2007. Deeranalysis 2006: Distance Measurements on Nanoscopic Length Scales by Pulse ESR. Springer.
25. Worswick, S. G., J. A. Spencer, …, I. Kuprov. 2018. Deep neural network processing of DEER data. *Sci. Adv.* 4:eaat5218.
26. Webb, B., and A. Sali. 2016. Comparative protein structure modeling using MODELLER. *Curr. Protoc. Protein Sci.* 54:5.6.1–5.6.37.
27. Wu, E. L., S. Jo, …, W. Im. 2014. CHARMM-GUI *Membrane Builder* toward realistic biological membrane simulations. *J. Comput. Chem.* 35:1997–2004.
28. Maier, J. A., C. Martinez, …, C. Simmerling. 2015. ff14SB: improving the accuracy of protein side chain and backbone parameters from ff99SB. *J. Chem. Theor. Comput.* 11:3696–3713.
29. Dickson, C. J., B. D. Madej, …, R. C. Walker. 2014. Lipid14: the amber lipid force field. *J. Chem. Theor. Comput.* 10:865–879.
30. Li, P., and K. M. Merz, Jr. 2016. MCPB.py: a Python based metal center parameter builder. *J. Chem. Inf. Model.* 56:599–604.
31. Firsch, M. J. 2016. Guassian 09, Revision E.01. Gaussian Inc.
32. Vosko, S. H., L. Wilk, and M. Nusair. 1980. Accurate spin-dependent electron liquid correlation energies for local spin density calculations: a critical analysis. *Can. J. Phys.* 58:1200–1211.
33. Lee, C., W. Yang, and R. G. Parr. 1988. Development of the Colle-Salvetti correlation-energy formula into a functional of the electron density. *Phy. Rev. B.* 37:785–789.
34. Rassolov, V. A., J. A. Pople, …, T. L. Windus. 1998. 6-31G* basis set for atoms K through Zn. *J. Chem. Phys.* 109:1223–1229.
35. Hutter, J., M. Lannuzzi, …, J. VandeVondele. 2014. cp2k: atomistic simulations of condensed matter systems. *Wiley Int. Rev. Computat. Mol. Sci.* 4:15–25.
36. Becke, A. D. 1988. Density-functional exchange-energy approximation with correct asymptotic behavior. *Phy. Rev. A.* 38:3098.
37. VandeVondele, J., and J. Hutter. 2007. Gaussian basis sets for accurate calculations on molecular systems in gas and condensed phases. *J. Chem. Phys.* 127:114105.
38. Van Der Spoel, D., E. Lindahl, …, H. J. Berendsen. 2005. GROMACS: fast, flexible, and free. *J. Comput. Chem.* 26:1701–1718.
39. McGibbon, R. T., K. A. Beauchamp, …, V. S. Pande. 2015. MDTraj: a modern open library for the analysis of molecular dynamics trajectories. *Biophys. J.* 109:1528–1532.
40. Peisach, J., and W. E. Blumberg. 1974. Structural implications derived from the analysis of electron paramagnetic resonance spectra of natural and artificial copper proteins. *Arch. Biochem. Biophys.* 165:691–708.
41. Xiao, Z., F. Loughlin, …, A. G. Wedd. 2004. C-terminal domain of the membrane copper transporter Ctr1 from Saccharomyces cerevisiae binds four Cu(I) ions as a cuprous-thiolate polynuclear cluster: sub-

femtomolar Cu(I) affinity of three proteins involved in copper trafficking. *J. Am. Chem. Soc.* 126:3081–3090.

42. Yang, Y., Y. Zhu, …, Y. Liu. 2019. Cuprous binding promotes interaction of copper transport protein hCTR1 with cell membranes. *Chem. Commun.* 55:11107–11110.

43. Shimberg, G. D., K. Ok, …, S. L. J. Michel. 2017. Cu(I) disrupts the structure and function of the nonclassical zinc finger protein tristetraprolin (TTP). *Inorg. Chem.* 56:6838–6848.

44. Kihlken, M. A., A. P. Leech, and N. E. Le Brun. 2002. Copper-mediated dimerization of CopZ, a predicted copper chaperone from Bacillus subtilis. *Biochem. J.* 368 (Pt 3):729–739.

45. Xiao, Z., and A. G. Wedd. 2010. The challenges of determining metal-protein affinities. *Nat. Prod. Rep.* 27:768–789.

46. Assafa, T. E., S. Nandi, …, E. Bordignon. 2021. Biophysical characterization of pro-apoptotic BimBH3 peptides reveals an unexpected capacity for self-association. *Structure.* 29:114–124 e113.

47. Giannoulis, A., Y. Ben-Ishay, and D. Goldfarb. 2021. Characteristics of Gd(III) spin labels for the study of protein conformations. *Methods Enzymol.* 651:235–290. https://doi.org/10.1016/bs.mie.2021.01.040.

48. Del Alamo, D., K. L. Jagessar, …, H. S. McHaourab. 2021. Methodology for rigorous modeling of protein conformational changes by Rosetta using DEER distance restraints. *PLoS Comput. Biol.* 17:e1009107.

49. Spicher, S., D. Abdullin, …, O. Schiemann. 2020. Modeling of spin-spin distance distributions for nitroxide labeled biomacromolecules. *Phys. Chem. Chem. Phys.* 22:24282–24290.

50. Ritsch, I., L. Esteban-Hofer, …, G. Jeschke. 2021. Characterization of weak protein domain structure by spin-label distance distributions. *Front Mol. Biosci.* 8:636599.

51. Schiemann, O., C. A. Heubach, …, G. Jeschke. 2021. Benchmark test and guidelines for DEER/PELDOR experiments on nitroxide-labeled biomolecules. *J. Am. Chem. Soc.* 143:17875–17890.

52. Raghuraman, H., S. M. Islam, …, E. Perozo. 2014. Dynamics transitions at the outer vestibule of the KcsA potassium channel during gating. *Proc. Nat. Acad. Sci.* 111:1831–1836.

53. Cai, Q., A. K. Kusnetzow, …, P. Z. Qin. 2006. Site-directed spin labeling measurements of nanometer distances in nucleic acids using a sequence-independent nitroxide probe. *Nucl. Acids Res.* 34:4722–4730.

54. Columbus, L., and W. L. Hubbell. 2002. A new spin on protein dynamics. *Trends Biochem. Sci.* 27:288–295.

55. Cornish, V. W., D. R. Benson, …, P. G. Schultz. 1994. Site-specific incorportaion of biophysical probes into proteins. *Proc. Nat. Acad. Sci.* 91:2910–2914.

56. Elgeti, M., and W. L. Hubbell. 2021. DEER analysis of GPCR conformational heterogeneity. *Biomolecules.* 11:778.

57. Ruthstein, S., A. M. Raitsimring, …, D. Goldfarb. 2009. Distribution of guest molecules in Pluronic micelles studied by double electron electron spin resonance and small angle X-ray scattering. *Phys. Chem. Chem. Phys.* 11:148–160.

58. Raitsimring, A. M., K. M. Salikhov, …, Y. D. Tsvetkov. 1974. Instantaneous diffusion in electron-spin echo of paramagnetic centers stabilized in solid matrices. *Fiz Tverd Tela+.* 16:756–766.

59. Spinello, A., J. Borisek, …, A. Magistrato. 2021. Compiuting metal-binding proteins for therapeutic benefit. *ChemMedChem.* 16:2034–2049.

60. Janos, P., A. Spinello, and A. Magistrato. 2021. All-atom simulations to studying metallodrugs/target interactions. *Curr. Opin. Chem. Biol.* 61:1–8.

61. Janos, P., J. Aupic, …, A. Magistrato. 2021. The conformational plasticity of the selectivity filter methionines controls the in-cell Cu(I) uptake through the CTR1 transporter. Preprint at BioRxiv https://doi.org/10.1101/2021.11.04.467269.

62. Sinani, D., D. J. Adle, …, J. Lee. 2007. Distinct mechanisms for Ctr1-mediated copper and cisplatin transport. *J. Biol. Chem.* 282:26775–26785.

63. Das, A., S. Chatterjee, and H. Raghuraman. 2020. Structural dynamics of the paddle motif loop in the activated conformation of KvAP voltage sensor. *Biophys. J.* 118:873–884.

64. Spinello, A., J. Borisek, …, A. Magistrato. 2021. Computing metal-binding proteins for therapeutic benefit. *ChemMedChem.* 16:2034–2049.

Biophysical Journal, Volume *121*

Supplemental information

Dynamical interplay between the human high-affinity copper transporter hCtr1 and its cognate metal ion

Gulshan Walke, Jana Aupič, Hadeel Kashoua, Pavel Janoš, Shelly Meron, Yulia Shenberger, Zena Qasem, Lada Gevorkyan-Airapetov, Alessandra Magistrato, and Sharon Ruthstein

Dynamical interplay between the human high-affinity copper transporter hCtr1 and its cognate metal ion

Gulshan Walke[a,#], Jana Aupič[b,#], Hadeel Kashoua[a], Pavel Janoš[b], Shelly Meron[a], Yulia Shenberger[a], Zena Qasem[a], Lada Gevorkyan-Airapetov[a], Alessandra Magistrato[b,*], Sharon Ruthstein[a,*]

[a] Dr. Gulshan Walke, Ms. Hadeel Kashoua, Ms. Shelly Meron, Dr. Yulia Shenberger, Ms. Zena Qasem, Dr. Lada Gevorkyan-Airapetov, Prof. Sharon Ruthstein
Department of Chemistry and the Institute of Nanotechnology and Advanced Materials (BINA), Bar-Ilan University, Ramat-Gan, Israel, 5290002
E-mail: Sharon.ruthstein@biu.ac.il
[b] Dr. Jana Aupic, Dr. Pavel Janos, Prof. Alessandra Magistrato
Department National Research Council of Italy (CNR) - Institute of Material (IOM) c/o International School for Advanced Studies (SISSA), via Bonomea 265, 34136 Trieste, Italy
Email: alessandra.magistrato@sissa.it

[#] Equal contributions
[*] Corresponding authors

Supporting Information

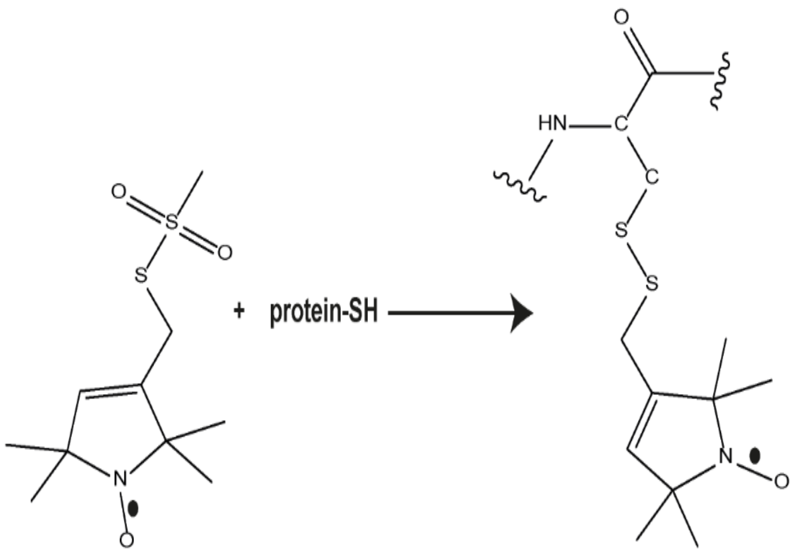

Scheme S1: Spin labeling scheme of cysteine residues with methanesulfonothioate (MTSSL) nitroxide radical.

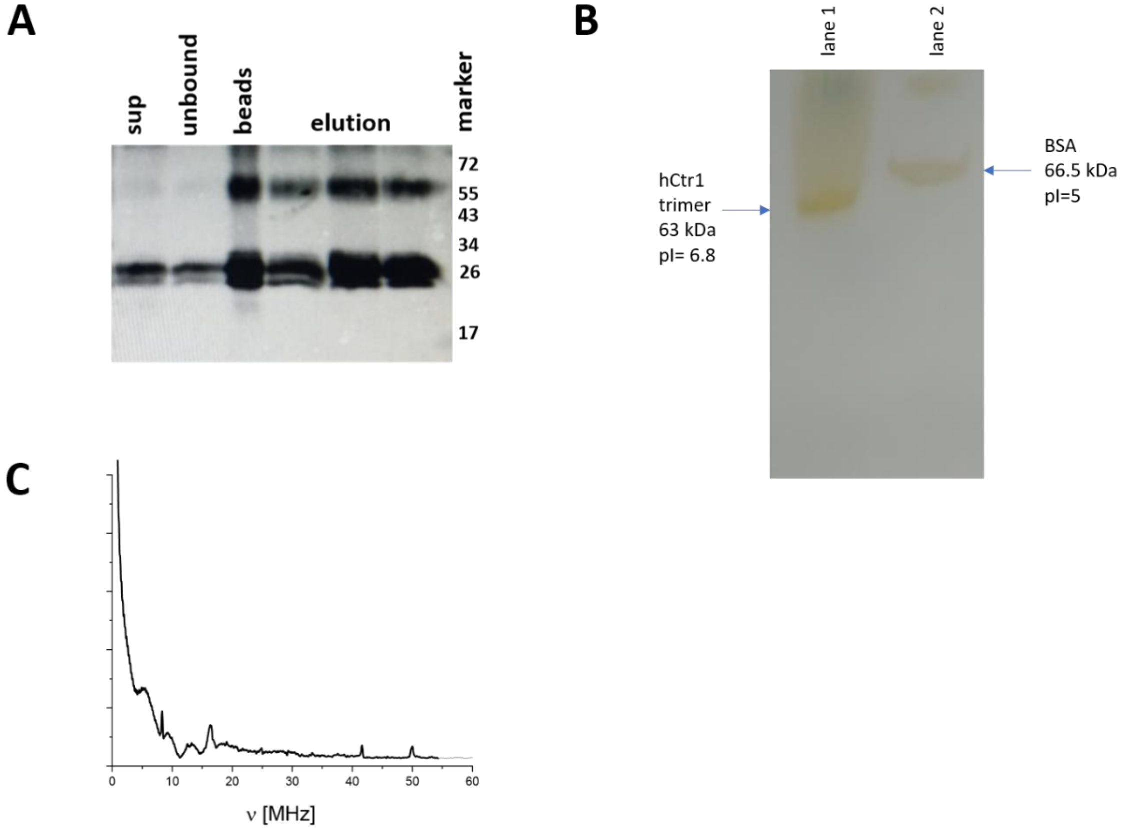

Figure S1: A. Western blot analysis of hCtr1 protein fractions probed. **B. Native-polyacrylamide gel electrophoresis.** Native-PAGE 12% glycine pH 8.5. Lane 1, the purified hCtr1 protein (MW of the monomer 21kDa, pI 6.8). Lane 2, the custom BSA protein (MW of the monomer 66.5 kDa, pI 5). **C**. **Q-band 3P-ESEEM FT spectrum** of Cu(II) hCtr1 (1:1 ratio). The experiment was carried out at 33.72 GHz, 11640 G, τ=180 ns, dt= 12ns. 600 points, Temp. =20 K.

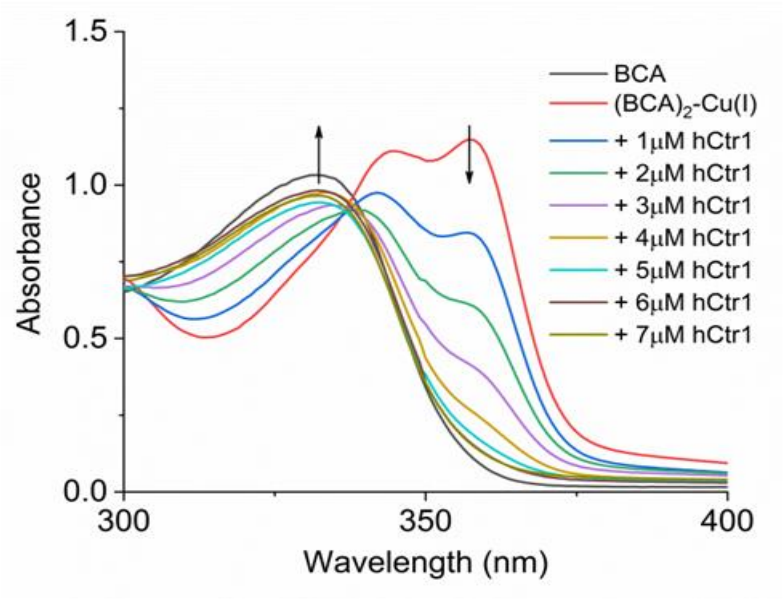

Figure S2. The UV-Vis spectra of the titration of hCtr1 to $(BCA)_2$-Cu(I) solution in UV region. The concentration of BCA and Cu(I) was 60 µM and 25 µM respectively to form 25 µM $(BCA)_2$-Cu(I) complex.

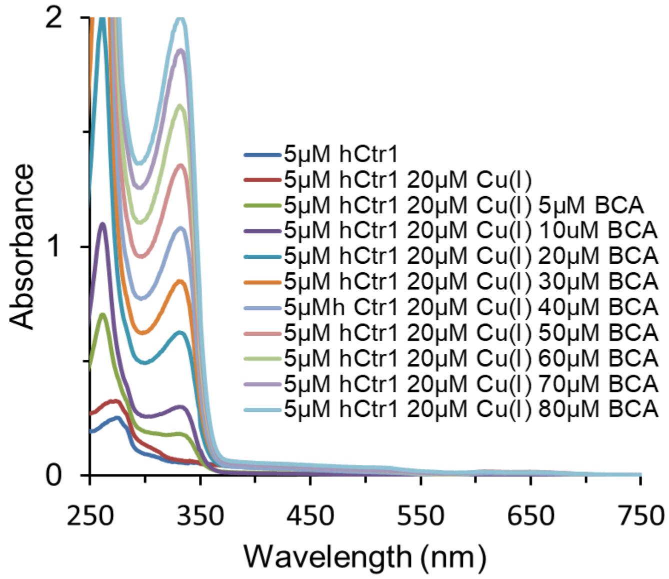

Figure S3. The UV-Vis spectra of the titration of BCA to hCtr1-Cu(I) solution in UV region.

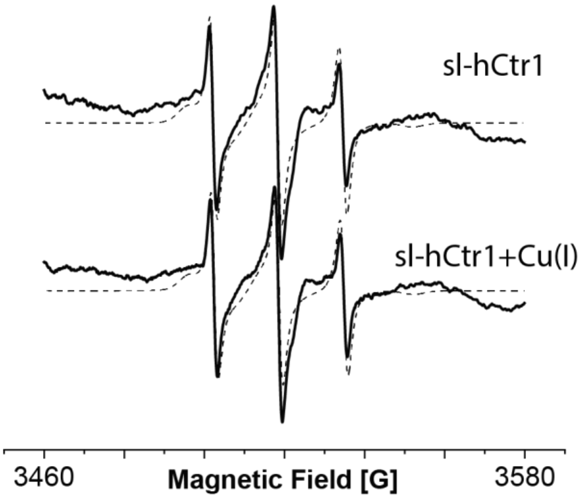

Figure S4. Room temperature X-band CW EPR spectrum of spin labelled (sl) wild type hCtr1 protein (named sl-hCtr1) in the absence and in the presence of Cu(I). The data was simulated using the easyspin program implemented in MATLAB, with g= [2.0087 2.006 2.0022], and A=[25 25 88] MHz.

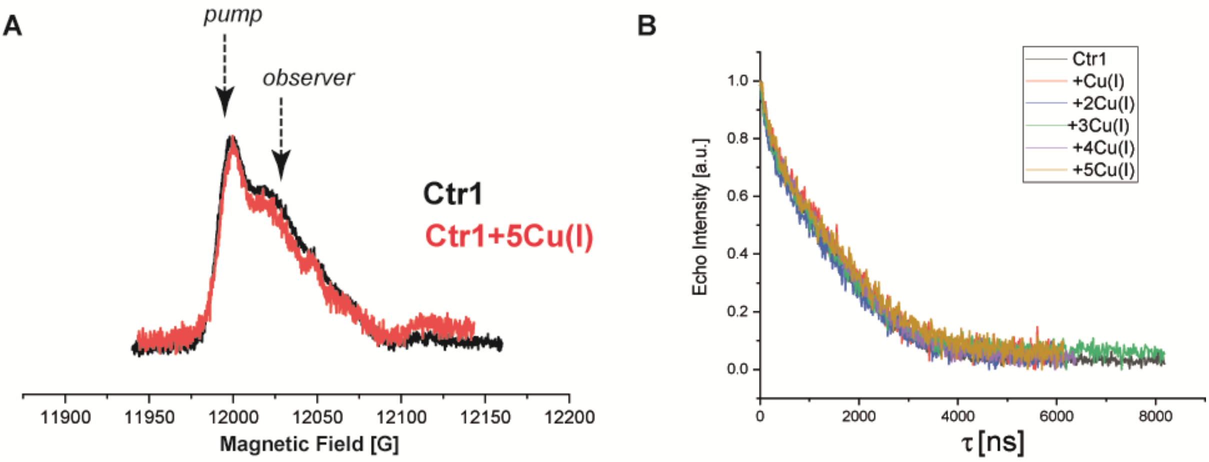

Figure S5. A. Two pulsed field sweep Q-band EPR spectra (50K) of wild-type spin-labeled hCtr1 in the presence and absence of Cu(I). **B**. two-pulse echo decay signals of spin-labeled hCtr1 at the absence and presence of Cu(I) ions.

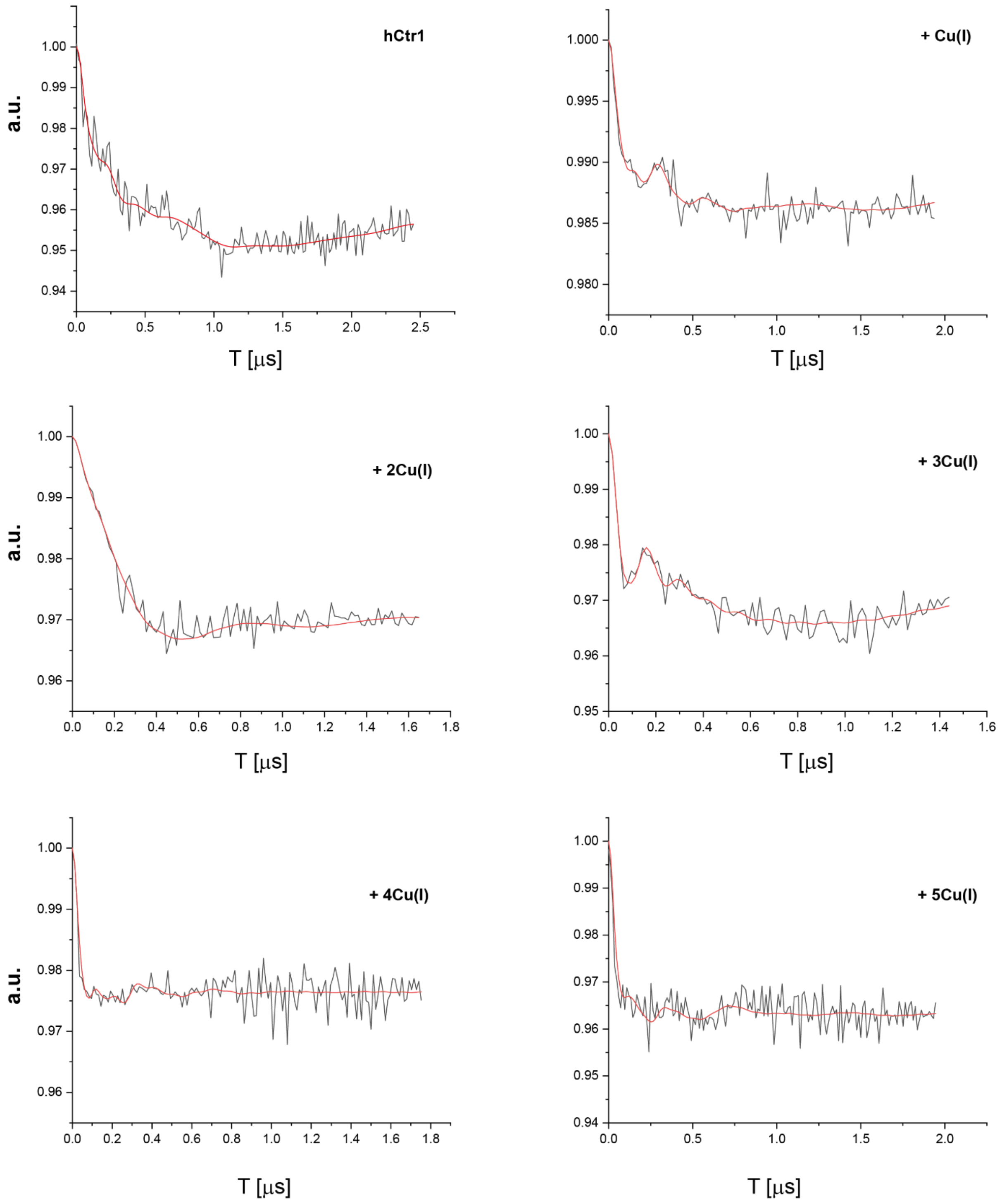

Figure S6. Q-band DEER time domain signals carried out at 50K of spin labelled wild type hCtr1 protein in the absence and in the presence of Cu(I) in Hepes buffer (0.1% Triton), pH 7.4

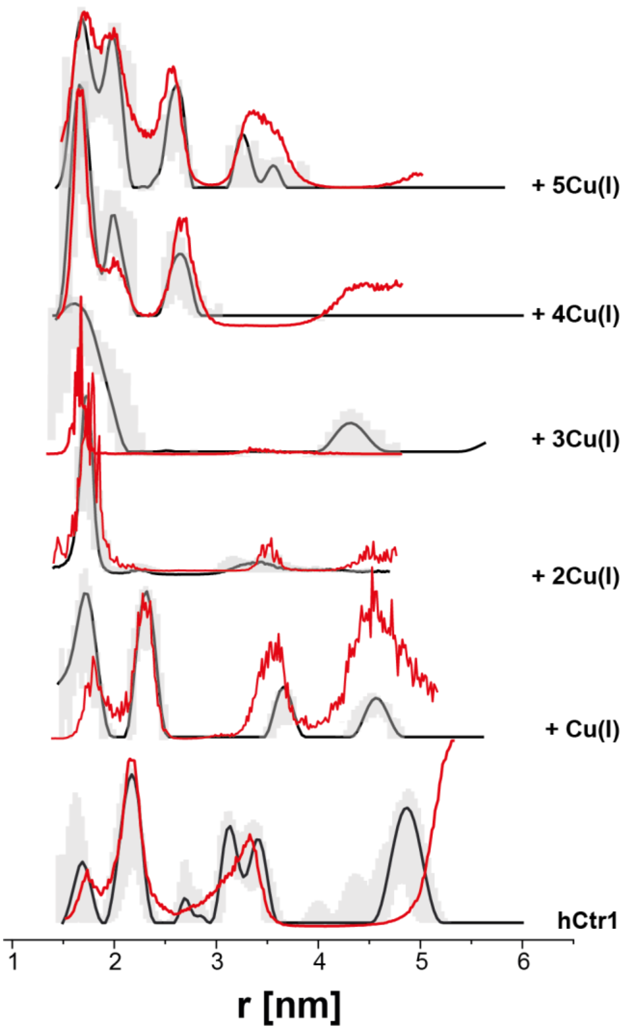

Figure S7. Q-band DEER distance distribution functions of spin labelled wild type hCtr1 protein. Black solid lines represent analysis using Tikhonov regularization, and red lines represent DeerNet analysis.

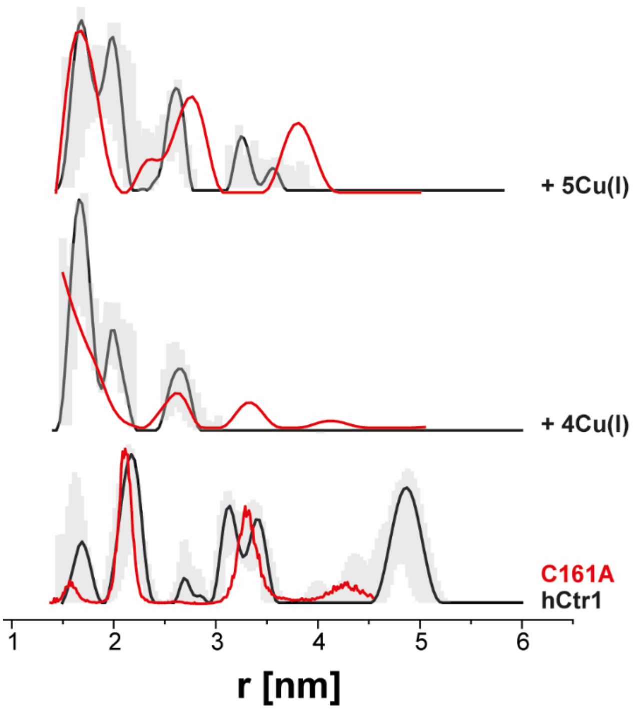

Figure S8. Q-band DEER distance distribution functions of spin labelled wild type hCtr1 protein (black solid line) and hCtr1_C161A mutant at various Cu(I) concentrations in Hepes buffer (0.1% Triton), pH 7.4.

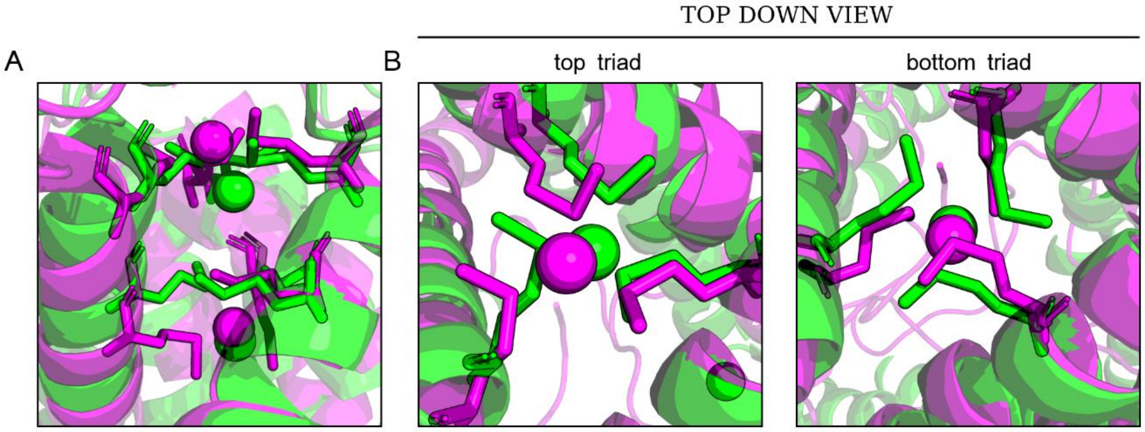

Figure S9. Comparison of the coordination geometry of Cu(I) ions bound in the selectivity filter in the crystal structure (PDBID:6m98) and in a representative structure extracted from QM/MM molecular dynamics simulation, shown in green and violet, respectively. **A**. The RMSD of the QM/MM MD optimized selectivity filter was 5.3 Å with respect to the crystal structure. RMSD was calculated using Met side chains and Cu(I) ions. **B**. Top-down view of the top and bottom Met triad (middle and right panel, repectively).

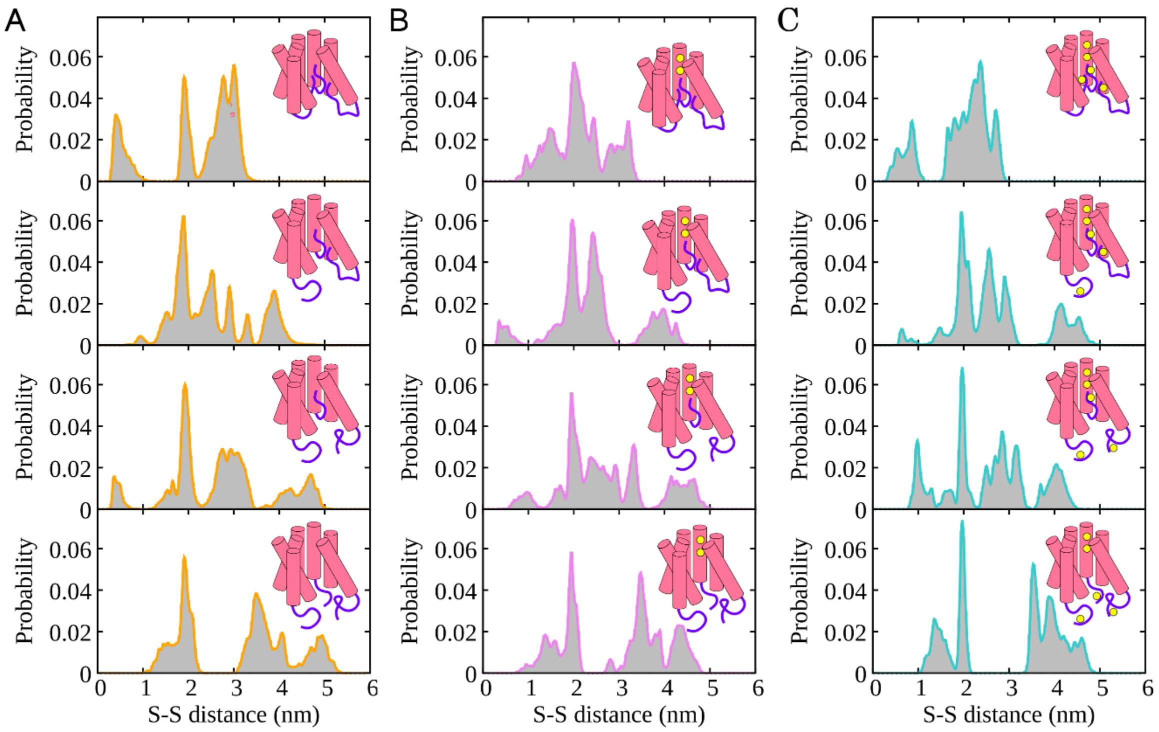

Figure S10. Distance distributions (nm) of Cys189 and Cys161 for all investigated hCtr1 models differing in the initial C-term tail conformation and Cu(I) loading. Distance distributions obtained for (**A**) apo hCtr1, (**B**) hCtr1 with two Cu(I) ions in the selectivity filter and (**C**) fully holo hCtr1 (with two Cu(I) ions in the selectivity filter and one Cu(I) in each C-terminal end). The cartoon shows the hCtr1 model with the transmembrane helices depicted in pink cylinder and each C-terminus depicted with a violet line. Copper is shown as a yellow circle.

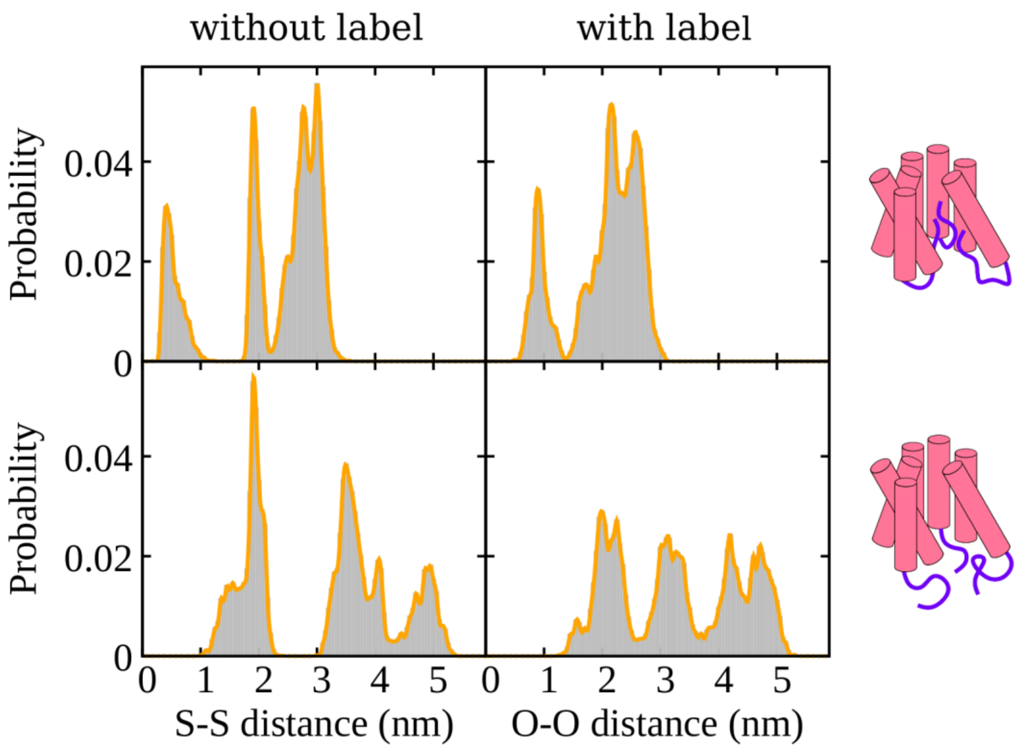

Figure S11. Impact of the MTSSL label on the distance distributions (nm) obtained from Molecular dynamics (MD) simulations. Left panels depict the distribution of distances between sulphur atoms present in Cys189 and Cys161 for the case where all three C-terminal tails are in the channel lumen (top) or cytosol (bottom). The MD simulations were performed in the absence (left panel) and in the presence of the MTSSL label attached to the Cys residues (right panel). Distance distributions were calculated between oxygen radical present in MTSSL. All simulations were performed for hCtr1 in the apo state.
